# Advancements in Testing Strategies for COVID-19

**DOI:** 10.3390/bios12060410

**Published:** 2022-06-13

**Authors:** Rabia Asghar, Madiha Rasheed, Jalees ul Hassan, Mohsin Rafique, Mashooq Khan, Yulin Deng

**Affiliations:** 1 Beijing Key Laboratory for Separation and Analysis in Biomedicine and Pharmaceuticals, School of Life Sciences, Beijing Institute of Technology, Beijing 100081, China; madiharasheed@bit.edu.cn; 2 Department of Wildlife and Ecology, Faculty of Fisheries and Wildlife, University of Veterinary and Animal Sciences-UVAS, Lahore 54000, Pakistan; jaleesulhassan@gmail.com; 3 Beijing Academy of Quantum Information Sciences, Beijing 100193, China; mmohsin8389@gmail.com; 4 Shandong Analysis and Test Center, Qilu University of Technology (Shandong Academy of Sciences), Jinan 250014, China; khan1985@sdas.org

**Keywords:** SARS-CoV-2, coronavirus, variants, immunoassays, diagnosis

## Abstract

The SARS-CoV-2 coronavirus, also known as the disease-causing agent for COVID-19, is a virulent pathogen that may infect people and certain animals. The global spread of COVID-19 and its emerging variation necessitates the development of rapid, reliable, simple, and low-cost diagnostic tools. Many methodologies and devices have been developed for the highly sensitive, selective, cost-effective, and rapid diagnosis of COVID-19. This review organizes the diagnosis platforms into four groups: imaging, molecular-based detection, serological testing, and biosensors. Each platform’s principle, advancement, utilization, and challenges for monitoring SARS-CoV-2 are discussed in detail. In addition, an overview of the impact of variants on detection, commercially available kits, and readout signal analysis has been presented. This review will expand our understanding of developing advanced diagnostic approaches to evolve into susceptible, precise, and reproducible technologies to combat any future outbreak.

## 1. Introduction

**Figure 1:**
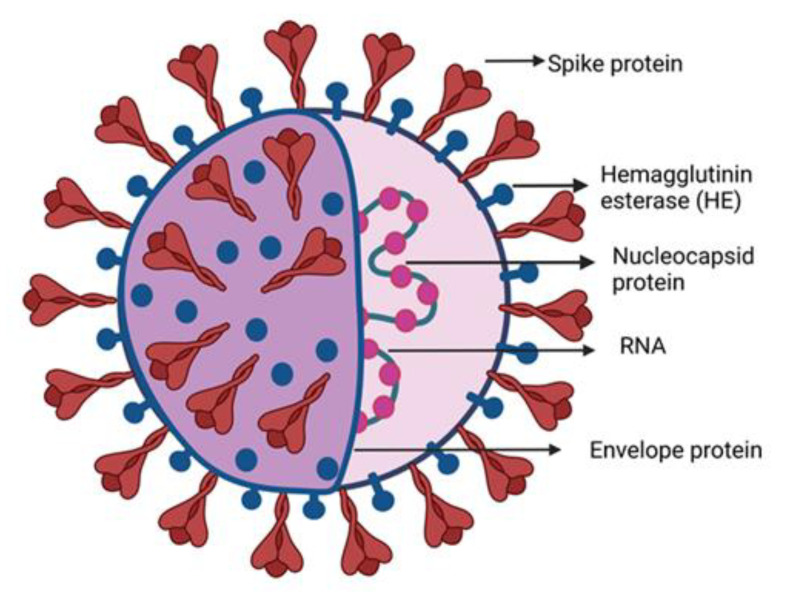
SARS-CoV-2 structure diagram. The majority of building proteins include spike (S), membrane (M), envelope (E), and nucleocapsid (N). The viral envelope and a lipid bilayer derived from the host cell membrane contain the proteins S, M, and E. The N protein binds to the viral RNA at the virion’s core.

The human coronaviruses causing fatal respiratory diseases were first observed in 1960 as the common flu [1]. The two pathogens SARS and MERS from the past were found lethal after attacking the respiratory tracts of the patients leading to nosocomial outbreaks [2,3]. Phylogenetic analysis reveals that bats are the natural reservoirs of SARS and MERS with the intermediate host Asian Palm civet and dromedary camels [4,5]. Severe acute respiratory syndrome corona virus 2 (SARS-CoV-2) emerged from the animal market in Wuhan, China. A sizeable (100–160 nm) group of spherically sensitive, non-segmented SARS-CoV-2 can infect both animal and human viruses [6,7,8,9]. The virus bypassed from the lineage B of coronaviruses due to the proteases breakdown at the receptor binding site of host cells, facilitating the transmission from animal to human [10,11]. SARS-CoV-2 belongs to the family Coronaviridae, subfamily Orthocoronavirinae, and order Nidovirales, which is further subdivided into four genera alpha (α), beta (β), delta (δ) and gamma (γ) CoVs, respectively. The lineage B of β-CoVs has three more lineages: A, C, and D [12,13]. The family contains the 10 deadliest human-borne viruses. For example, the death rates for SARS-CoV and MERS-CoV are 10% and 36%, respectively. The α- and β-coronaviruses have their origin in mammals including camels, pigs, bats, and rodents, while the other two δ and γ-coronaviruses infect birds and mammal whales. Generally, these viruses infect the respiratory and digestive tracts of their hosts [14]. SARS-CoV-2 contains a single strand RNA of ≈30 kb which has a cap-like structure at 5′ and a poly-(A) tail at the 3′ end. In the Figure 1 the structural elements of the SARS-CoV-2 are shown. The genomic RNA is surrounded by the basic structural protein, giving it a crown-like appearance. The nucleocapsid protein (N-protein) enveloped the viral genome in a helical pattern, while the membrane protein (M-protein) incorporated with the inner nucleoproteins to form a basic structure [13,15,16,17,18].

The order of the SARS-CoV-2 genome is 5′-cap structure replicase (open reading frame 1/ab)–structural proteins linked with a spike–envelope–membrane-nucleocapsid (N)—3′ poly (A) tail is similar to the other β-CoVs. Furthermore, the variants of SARS-CoV-2 have been observed in several countries and reported by the WHO. The variants are named after the first reported date or the changes in the sequence of the amino acids. The WHO reports categorized the variants as “variants of interest” (VOI) and the “variants of concern” (VOC), along with the recommended actions that need to be taken by the state after its identification. The VOI is said to be the SARS-CoV-2 isolates with different genomic and phenotypic changes compared to the reference genome, while VOCs are the VOIs that have a demonstrable increase in transmissibility and virulence without effective control by current public health measures [19]. The transmissibility of the pathogen is its strength to invade a population and the measurement of the required strength to eliminate the spread of the pathogen. The transmission potential of the pathogen is defined by its reproduction rate (R₀) [20]. The reproduction rate of the SARS-CoV-2 is up to 3.6 comparatively higher than that of SARS-CoV (R₀ = ~3.0) and MERS-CoV (R₀ = ~1.5) [21]. The presence of more cross-protective epitopes of S-protein than N-protein in SARS-CoV-2 makes it more contagious than SARS-CoV and MERS-CoV [22]. The viral genome after entry into the host cell served as a template to translate polyprotein ORF1/ab to build 16-nonstructural proteins and proteolytically cleaved to perform their putative functions [18]. These putative functions are to serve as replication and transcription complexes (RTCs) for the further generation of viral copies or as templates as a negative-sense RNA intermediate to produce positive-sense strand and sub-genomic RNAs by RTCs [23,24]. The incubation period of the original strain of SARS-CoV-2 is from 2 to 5.6 days to the maximum of day 12, or maybe patients do not experience any symptoms. Comparatively, the variants of SARS-CoV-2 showed up the symptoms faster [25]. The SARS-CoV-2 due to its high transmissibility, shorter incubation period, and highly potent variants has led to the ongoing pandemic becoming a global challenge. In addition, the mutations that have a phenotypic expression of high transmissibility and virulence must impact the diagnosis. The increasing number of COVID-19 patients is causing loss of lives and economic costs. The simple and fast monitoring of viral particles in the bio-fluids can help to diagnose the healthy/unhealthy conditions of a person and ensure early therapeutics. In this review, different techniques including molecular-based detection, serological testing, bio-imaging, and biosensors used for the diagnosis of COVID-19 in biological samples were discussed. In the beginning, the factors, such as the person’s travel history, auxiliary examinations, or imaging, including CT scan or chest X-rays, were to be considered for the diagnosis. Subsequently, DNA sequencing and reverse transcription-polymerase chain reaction (RT-PCR) were used for the diagnosis of COVID-19 [26]. DNA sequencing is also significant for the monitoring of SARS-CoV-2 variants [27]. During the current pandemic, the unprecedented situation comprised of many challenges such as detection at the individual level at mass level screening. The basic testing procedures that are being followed are molecular assay and immunoassays. These testing techniques are modified to meet current needs. Many test formats have been established, such as lateral flow immunoassay [28], ELISA [29], dipstick [30], and cartridge-based platforms [31] to enhance the testing specificity and sensitivity. The detection of SARS-CoV-2 has been through many interlink-modified methods. The challenges these approaches faced in the analysis of different variants are also described. The future directions for the possible solutions to the challenges in these techniques for commercially advanced applications of current methods are included. This review will be of great interest to the researchers working in the same domain, and it will serve as an informative tutorial tool for researchers from other fields and beginners. This study will explore the possibility of easy surveillance with in vitro diagnostic devices for the detection of COVID-19 to prevent its fast spreading.

## 2. Detection Techniques of SARS-CoV-2

Moreover, detection strategies have been proposed based on different degrees of specifications. Each procedure followed the single or multiple targets related to the novel virus. Table 1 presents the conventional detection methods based on commercialized and non-commercialized techniques with advanced modifications.

### 2.1. High Throughput Sequencing

Next-generation sequencing (NGS)-based strategies are used to trace the evolutionary history and to investigate the chain of transmission of disease during the outbreak. The complete genomic sequence of SARS-CoV-2 was released in January 2020 [52]. The sequence has over 82% similarity to those in SARS-CoV and bat SARS (SL-CoV) [53]. Nasopharyngeal swabs from the respiratory tract were used to analyze the viral load [54]. The NGS proved significant for the diagnosis of severe infections or the patient that carries pathogens of unknown origin [55]. Recently, variants of SARS-CoV-2 have also been traced by SNP genotyping [56]. This ultrasensitive high-throughput sequencing method is expensive, time-consuming, and dependent on stringent laboratory equipment, limiting its use in the clinical diagnosis of COVID-19. Therefore, a cost-effective and fast testing procedure is needed to develop for further investigations.

### 2.2. Imaging

Initially, imaging techniques were used to diagnose and observe the severity of COVID-19 infection. The Computed Tomography (CT) scan images of 63 patients were taken from a hospital possessing some variant results of affected lungs images. The symptoms observed included affected lobes, patchy consolidation and fibrous stripes, and some complex irregular solid nodules while enlarged, which varied differently in different patients. This imaging was a supportive method for the diagnosis before the submission of the genomic sequence of the virus [57]. However, this high-resolution CT scan is an indicative but non-confirmative method due to the lack of difference between viral and non-viral infection of respiratory tracts, which limits its diagnostic applications [58,59]. So, there is a need to diagnose the disease at the molecular level with more specification and authenticity at the individual level before further treatments.

Advanced imaging optical coherence tomography (OCT) has been applied for the diagnosis of acute respiratory failure. This real-time 3D imaging technique is used to have a visual demonstration of alveolar compartments and air pathways. The potential application of OCT for COVID-19 patients can be devised to observe the lungs of the patients [60]. Alternatively, the application of photoacoustic imaging has potential for the analysis of the inflammatory markers in the lungs for the clinical diagnostics [61].

### 2.3. Microarrays

Nucleotide-assisted microarray-based detection strategies have been adopted for the detection of newly invaded coronavirus into the SARS family in the past years. The 60 mer oligonucleotides were designed for the detection of coronavirus from the SARS family. Thirty designed oligonucleotides of 60 mer (TOR2) were able to detect the whole genome of the submitted new coronavirus strain [62]. In another study, a microarray was designed for the detection of a mutated spike gene (27 single nucleotide polymorphism), which was correlated to the pathogenicity and epidemiology of the disease by SARS-CoV. The designed SNP DNA microarray served as the detection tool with ≈100% accuracy. A non-fluorescent-based low-density oligonucleotide assay was utilized for the point-of-care testing of SARS-CoV-2. This approach has a comparable sensitivity with RT-PCR with the limit of detection of 15.7 copies per reaction for the other HCoVs [63]. Similarly, a more sensitive platform was used for the diagnosis of respiratory infection and MERS that may also be useful for the detection of SARS-CoV-2 [64].

### 2.4. Molecular Assay-Based Diagnosis

Nucleotide-based detection methods are considered as most reliable for individual-level testing. The developing kits have been designed in many ways of targeted sites, sets of primers, the principle of the test, and final signal readout in a way to be more specific.

#### 2.4.1. PCR-Based Methods

The polymerase chain reaction (PCR) methods applied for nucleic acid amplification testing include a list of delicate steps such as sample collection and its transport, viral extraction, amplification, and signal readouts. The primary step of sample collection is performed by the swabs, or forks containing CDC recommended materials nylon or polyester fibers on a plastic stick over wooden shafts or, even better, the calcium alginate sticks to avoid contaminations. At the beginning of the pandemic, a short supply of swabs created a bottleneck. To overcome this challenge, 3D-printed nasopharyngeal swabs were designed [65]. A clinically validated medium, phosphate buffer, was used as stable transportation of the viral medium. This can keep samples stable for up to 18 h for qPCR testing without compromising the detection of N, S, and Orf1ab targets [66].

After pre-analytical steps, analytical accuracy depends on the nucleic acid extraction before amplification and final signal readouts. False-negative testing also increases if the contaminated or low viral load is further processed. Conventionally, organic phenol-chloroform extracts nucleic acid by simple degradation of protein and non-nucleic acid parts by the action of SDS and proteolytic enzyme K [67]. In contrast, a more efficient acid-pH method is utilized for the nucleic acid extraction of SARS-CoV-2 [68]. In this method, the sample is directly incubated with proteinase K and heated at 98 °C for 5 min [69]. Subsequently, the PEARL (precipitation enhanced analyte retrieval) is performed to break down the non-nucleic acid components by a lysis solution which yields a precipitate of alcohol-based nucleic acid [70]. Due to the use of various sample reagents and centrifugation steps that elute bonded nucleic acid to the column supports, silica-functionalized magnetic microbeads were applied on a testing LionX system platform to devoid the elution step.

The extracted genome is then added to the target gene primer, probe, and a master mixture. Amidst other amplification, the qRT-PCR is the most reliable clinical testing for detecting infectious pathogens alternative to Northern blotting-based assays [68]. The previously reported coronaviruses were detected by the same method [70,71,72,73]. In the prevailing period, RT-PCR is a gold standard for several facts such as the specificity of the particular target strain of the SARS-CoV-2 without cross-reactivity with preceding human CoVs. The key to the specificity is primer-probe binding to the target gene; a comparative evaluation of the sensitivity of different primer-probes sets was checked according to the target gene (N, N2, and N3). RNA isolation were performed by using Vero cell culture, QIAamp Viral RNA Mini Kit (#1020953, Qiagen, Hilden, Germany) and RT-ddPCR was performed using primers and probe published by the Chines CDC. The target genes Orf1ab (Beijing, China), NIID_2019-nCoV_N (Tokyo, Japan), and 2019-nCoV-N2 (US-CDC) were found more sensitive to apply for the clinical analysis; later, excluding the target N3 did not affect the sensitivity of the assay [74,75]. The multiple target detection makes it more accurate and high throughput to apply on multiple parallel assays (384-wells plate).

Being most frequently available, it is a highly significant and sensitive, direct, and rapid procedure in routine practice [76]. The amplification of cDNA through PCR proceeding to quantification and detection was performed on conventionally accepted agarose gel or DNA sequencing procedures [77,78]. Despite some clinical limitations such as time consumption and dependency on the instrument or trained workers, in the past studies, a single-tube RT PCR method was applied for the identification of respiratory pathogens such as HCoV-OC43 and HCoV-229E targeting the gene Orf1b [79,80,81]. In addition, RT-qPCR has other disadvantages of potential biological safety risks, nuclear extraction, and sophisticated laboratory equipment such as biosafety cabinets, which are often available in a few central laboratories, and sample transportation and processing to the laboratories [80].

According to FIND diagnostics, a total of 435 SARS-CoV-2 RT-PCR-based kits have been designed, and among these, 235 kits have been approved by the Food and Drug Administration (FDA) for the commercial applications. Most of these have multiplex targets to attain more sensitive results. More targets serve as the templates to transcribe into complementary DNA, which further act as templates for the extension. During extension, the Taq polymerase released the reporter dye from the 3′quenching dye after cleaving annealed probes, which increases the fluorescence relevant to the amplified part. The companies and researchers are modifying the techniques by improving various steps of the molecular assay. Unique molecular testing is introduced in less than 13 min ID Now ^TM^ by Abbott, based on the rapid isothermal amplification of the target pathogen to generate a short segment of the target pathogen, which is later recognized by its fluorescent probes. Moreover, various modifications such as the automated extraction of nucleic acids, amplification procedures, and better signal readouts can improve the test run time, minimize the cost, process a large sample, reusability, and multiplex detection.

##### Isothermal Amplification

Isothermal amplification is an excellent alternative to PCR amplification to avoid highly expensive thermal cyclers, and it is a rapid and efficient amplification process to amplify nucleic acid sequences at a constant temperature. Amplicons produced by this procedure are far better at producing nucleic acid base nanomaterials to utilize in biomedicines, biosensing, and bio imaging. For biosensing DNA, RNA, cells, peptides, some molecular, and sub-molecular species are the selected targets. According to reaction kinetics, here, we discuss the following three categories.

##### RT-Loop-Mediated Isothermal Amplification (LAMP)

LAMP assays have been used in many studies for the detection of SARS-CoV and also for other human coronaviruses, particularly HCoV-NL63 [82,83]. Quantitative RT-LAMP tests were designed for the early analysis of SARS-CoV [84]. For instance, a rapid, reliable, reusable, and robust point-of-care RT-LAMP was introduced for the detection of SARS-CoV-2, which is 12 times more sensitive and 10 times less expensive than the conventional RT-PCR [84]. The RT-LAMP assay has several advantages over RT-PCR, including direct detection without the laborious step of RNA extraction [84,85], lack of cross-reactivity with other respiratory pathogens [86], and colorimetric and fluorescent-based signal detection within 20–30 min at a temperature of 63–65 °C [87,88]. The improved and more specific method was used for the detection of MERS-CoV in past studies to overcome the problem of turbidity due to the production of pyrophosphates (white precipitates) during the polymerization reaction. The fluorescent dyes intercalate in double-stranded amplicons, which cause non-specificity to produce a signal from primer dimerization or non-primer involvement [89]. Therefore, a temperature-specific DNA amplification LAMP method and quenching probes were introduced to track the true signal in the reaction for the specific diagnosis of MERS-CoV [90]. A similar method has been utilized for the detection of SARS-CoV-2 [38,91]. Yet, this method required a fluorophore and a quencher for labeling, because the toehold is located at the end position of the hairpin stem. So, it may cause an improper quenching due to high background signals. In addition, these molecular techniques have limitations for full-length genome analysis [87,88]. In recent advancements, LAMP has been improved by introducing artificial intelligence-based results interpretation. A smart palm top diagnostic device was designed to produce automatic image and algorithmic data processing through artificial intelligence. Such devices have improved the run time of tests and pH-dependent colorimetric detection. The specificity, sensitivity, and reliability of the test procedure were performed on 200 suspected patients and were provided by NHS to validate against the target RdRp [92].

##### One-Pot Enzyme-Free Isothermal Amplification

**Figure 2:**
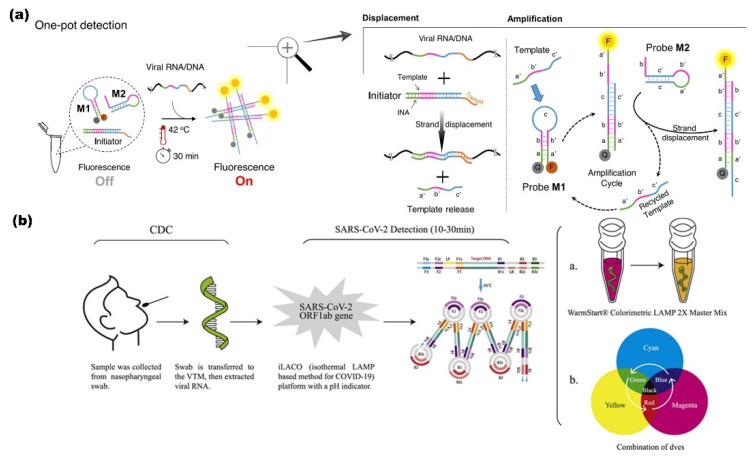
(**a**) NISDA assay (non-enzymatic RT-LAMP); components of reaction mixture include DNA duplex and two DNA probes (M1 and M2). Template displacement is triggered by toehold upon detecting the target, followed by a cascade of sequential amplification of the signal. Quenched 6-FAM fluorophore (bhq-1) restores fluorescence upon detecting target (viral RNA/DNA) after 30 min at 42 °C. Letters labels represent domains, while prime labeled domains donated complementary sequences Readapted with permission [93]. Copyright © 2021, The Authors. (**b**) Colorimetric sensor based on iLACO system. I: LAMP in Master Mix II: Combining different dyes. Reproduced with permission [94]. © 2021 The Authors. Published by Elsevier Ltd.

A one-pot enzyme-free isothermal amplification method was developed for the ultrafast (<20 min) analysis of clinical samples. Non-enzymatic isothermal strand displacement (NISDA) assay (Figure 2a) involved a two-step displacement and amplification process. In this method, a DNA duplex is used as an initiator, which converts the viral RNA into short DNA by using intercalating nucleic acid (INA) technology. The next amplification step followed the initiation of a series of reactions to unwind DNA beacon structures (Probe M1 and M2), imposing the striking increase in fluorescence of M1. Here, in contrast to TMSD assays, the toehold of M1 present at the c domain of the loop region facilitates the proper quenching. NISDA enabled SARS-CoV-2 with a limit of detection of 10 copies/μL [93].

##### RNA Auto Cycling

The auto cycling process can effectively detect the RNA directly from the raw cell lysate without the RNA extraction step. The detection of SARS-CoV-2 from pharyngeal swabs without the RNA purification step is widely applied [91]. The visual amplification (Figure 2b) and detection of SARS-CoV-2 using a combination of dyes instead of a commercially available single dye indicator to enhance the signal readouts with a broad spectrum of dyes improves the rapidness of the assay for the multiple target detection [94].

### 2.5. CRISPR Based Detection

**Figure 3:**
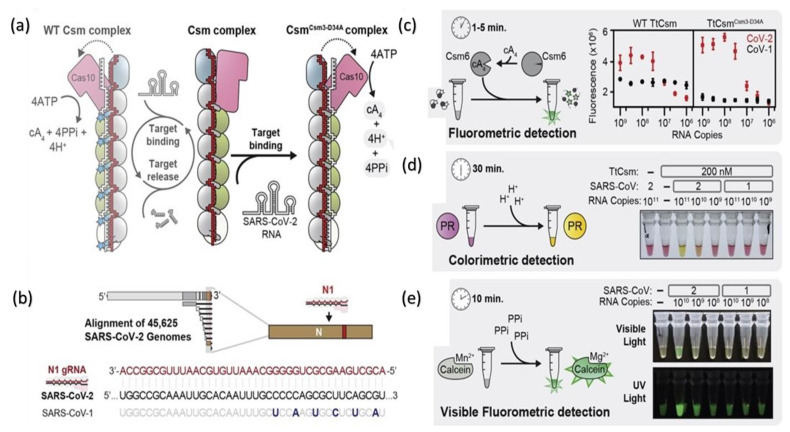
(**a**) A schematic representation of CRISPR Csm complex type III (Thermus thermophilus) consisting of a CRISPR-RNA (red) and a set of 5 stoichiometrically unequal proteins [Cas 101 (pink), Csm41 (blue), Csm36 (gray), Csm24 (green), Csm51 (white)]. Enzymatic cascade (Cas10-polymerase, Cas DNAase and Csm3 RNAase) activates due to the binding of CRISPR-RNA. Csm3 subunits cleave target RNA and the inactivation of Cas10. The complex RNase-dead is generated due to mutant TtCsm^Csm-D34A^. (**b**) SARS-CoV-2 genome and N1 region of CRISPR RNA (crRNA_N1_). (**c**) Fluorometric detection, a transcribed SARS-CoV-2, and N-gene of SARS-CoV-1. A non-sequence-specific ancillary nuclease, cyclic tetra-adenylate (cA_4_), activates the TtCsm6.RNA tether furnish link between a fluorophore (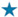) to a quencher (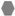). Mutant in the right graph showed a lower LOD (3-folds) than the wild in the left graph. (**d**) Colorimetric detection of SARS-CoV-2 by mutant N1 complex by a dye phenol red (a pH-sensitive dye) incubated at 60 °C for 30 min. (**e**) Visible fluorometric detection by the mutant N1 complex using calcein, incubated for 60 min at 60 °C [102].

A nucleic acid-based detection by clustered regularly interspaced short palindromic repeat (CRISPR (Cas-9, Cas-12a, Cas-12b, and Cas-13)) is a gene-editing technique that aids researchers to add, delete, or modify the genome at a required specific domain on the gene map [95,96,97,98]. A previous study implanted to delete the RNA-based viruses by using Cas-13 from mammals [99]. The same procedure was used for the detection of the dengue, Zika virus ssRNA genome. The SHERLOCK protocol is a specific and highly sensitive enzymatic reporter unlocking for a portable and multiple nucleic acid base detection from clinical samples. The whole essay includes a series of reactions of pre-amplification of DNA or RNA and subsequent enzymes followed by Cas-12 or Cas-13 mediated detection through colorimetric and fluorescent signals. The observed run time of the assay is less than 15 min, and total signal readouts are provided in less than 60 min [100]. Moreover, Cas12a, Cas12b, and Cas 13a nucleases cleavage activity are to develop point-of-care testing of SARS-CoV-2 in different studies’ workflows schemes after modifying according to required setups. A more sensitive RT-PCR assay detection was performed (10 copies/reaction in 40 min) by RPA-mediated DNA amplification and signal amplification by CRISPR-Cas-12a [39]. Another CREST, Cas-13-based detection was performed on low-cost thermocyclers and accessible enzymes such as Taq polymerase based on fluorescence signal amplification to detect ten copies/μL [100]. All-in-one CRISPR-Cas 12a (AIOD-CRISPR) assay was modified without pre-amplification steps of RNA directly; all incubated components in a single reaction with 4.6 copies of SARS-CoV-2 within 40 min [98]. Regardless of the advantages, the procedure has some limitations, such as expertise dependent on the preparation of reaction components and reaction steps such as protein purification and RNA extraction. Moreover, multistep amplification and digital quantification may affect precise testing. Another rapid (≈30 min), inexpensive, and easy to handle diagnostic technique introduced the CRISPR-Cas-12 platform as DETECTR (DNA endonuclease-targeted CRISPR Trans reporter for the detection of viral infections) [97,101]. DETECTR is based on the lateral flow assay alternative to PCR testing, with 95% positive predictive agreement and 100% negative predictive agreement for the viral detection in <40 min [39]. It is a rapid and multiplex sequence-specific viral RNA detection using RT-LAMP coupling with T7 transcription and Csm-based (Figure 3) detection of SARS-CoV-2 in less than 30 min with an attomolar sensitivity and high specificity. The assay can be applied for the detection of variants and other pathogens as well. The reaction modification is possible to a “one-pot diagnostic” by redesigning primers at 55 °C to enhance the efficiency of T7-Csm at 65 °C [102]. In a recent study, CRISPR-Cas13 has designed a comprehensive investigation of notable viral RNAs and a broad spectrum observation for antiviral targets of 16 families of human–animal infectious viruses by applying in silico analysis in a ViPR database [96]. Pre-amplification of RNA limits its high-throughput application. Amplification-free CRISPR-Cas-13a is based on mobile phone microscopy that directly detects viral RNA from the nasal swabs. Moreover, the combination of crRNA may increase the sensitivity to the attomolar range (≈30 copies/μL). Multiple target recognition by crRNA avoids the detection conflicts raised after the variants interference [103]. Another detection strategy is CRISPR-Cas-13d PAC-MAN (prophylactic antiviral CRISPR in a human cell) RdRp and N-antigen of the SARS-CoV-2 and also worked on Influenza A.

### 2.6. Limitations of Molecular Diagnosis

It is hard to validate all molecular diagnostic techniques in a specific way. There are several pre-analytical and analytical factors responsible for false-negative testing such as low viral loads, viral shedding time, the sample collection site (nasal swabs, upper respiratory tracts, or lower respiratory tract), and the time of sample collection day after on-set of symptoms (0–7, 8–14 or ≥15 days) [104]. For example, samples from lower respiratory tracts and the sputum have the highest positivity rates of 93% and 72%, respectively, while samples taken from nasal swabs, upper pharyngeal, feces, and blood have corresponding low positive rates of 63%, 32%, 29%, and 1%. A systematic study reported up to 54% of initial false-negative RT-PCR testing rates [105]. In another study conducted in New York, the clinical performance of SARS-CoV-2 was evaluated by retesting the negatively tested patients on the same day. An increase in positive rate from the negative rate was observed due to inadequate sample, incorrect sample collection, and stochastic bias from the low viral load [106]. The detection sensitivity of RT PCR assay is varied from different target regions because highly conserved region targets and multiple targets could be applied to reduce invalid false-negative results. Mutations in a primer region also affect primer-probe binding [107]. A one-step quantitative assay has 10 times greater sensitivity for the N-gene over open reading frame 1 ab. At the same time, CDC proposed that the set of primer probes for the N-gene (N1, N2, and N3) and E-gene have better sensitivity for N1, N2, and the E-gene over N3 and RdRp. The impartial application of metagenomic assay and unauthentic information on possible coinfections would urge the researchers to develop improved treatment. Moreover, CRISPR-based strategies continue to grow, but their extensive clinical application for the rapid detection of pathogens can be restricted in limited resources settings. While the sensitive PCR tools are not available or lack suitable sample collections, a significant viral load for a small genetic expression of the virus hampers their applications. Moreover, the turnaround time of sequencing is far greater than the widely accepted techniques. Metagenomics, RT-PCR and CRISPR hold great promise as a distinct diagnosis, especially in patients carrying novel respiratory pathogens. All these three methods involve a 1 h RNA extraction step. The remaining RT-PCR and CRISPR workflows are also designed to prepare the library, while the mNGS flow has several parts with different periods of time: 3 h for library preparation, 18 h for mNGS sequence and 3 h for data analysis. Therefore, the turnaround time (TAT) for RT-PCR, CRISPR and mNGS is about 3, 2, and 24 h, respectively. Moreover, RNA extraction is a must by depending upon biohazard safety labs to handle sensitive testings. Despite efficient diagnostics, 1-step detection devices or biosensors for point-of-care testing is the need of the hour [55,108]. Recently, portable CRISPR-based COVID testing miSHERLOCK was introduced by combining two unrelated CRISPR nucleases (Cas 13 and Csm 9) in a tandem assay on a portable microfluidic chip [109]. A minimum dependency on the instrument, alternative to PCR testing, and portable chip with sensitivity (100 copies/µL) within 20 min can be advancement toward molecular-based testing.

### 2.7. Signal Readout Methods

Signal readouts are the quantification steps of the reaction yield for an efficient diagnosis. These methods include optical, electronic, colorimetric, and electrochemical, following their different strategies. The means used to read signals varied according to the design. The possible means of optical signaling are naked-eye, color change, fluorescence emission, and optical reflections, which are usually carried on the platforms such as microfluidic devices, microplate readers, spectrophotometers, cartridges, strips, and cupids. Florescence and colorimetric-based optical signaling are the most applied strategies during the development of SARS-CoV-2 detecting kits. The conjugated fluorescent dyes nonspecifically bind to the double-stranded amplicons and non-specifically to the single-stranded target probe to quantify the amplification product visually. These signal readers are integrated electronically with smart gadgets or plate readers to measure fluorescence. The improvement in signal detection methods improves the sensitivity of the procedure [110]. The conventional signal readout method is highly delicate to interpret the final results. Conventionally, detected SARS-CoV gives one copy per reaction, while SARS-CoV-2 detected by fluorescent signal readout gives 100 copies/mL. A portable detection platform was developed by incorporating PCR with a smartphone [111]. Further CRISPR methods also used the modified DNA fragments with fluorescent probe quencher combos.

### 2.8. Serological Testing

Antibodies are the proteins that are produced in the body within 1–3 weeks of the infection. Combat to the disease depends upon the antigenicity (the ability of an antigen to induce an immune response) of the invaded pathogen and the host’s immunogenicity (responses from the host to produce antibodies). The challenges such as unavailability of RT-PCR, denaturation of viral RNA during sample collection and extraction steps, shortage of primers, and mutations in viral genomes urge us to use an alternative, cheaper testing method. Serological testing procedures are helpful to trace the contact and the extent of body response toward infection as well as conduct an epidemiological survey and identify past and post-infection responses of the body. An accessible sampling collection and transportation of blood samples taken from finger sticks or veins compared to the PCR testing sample collection of mucus from the nasopharyngeal tracts is more sensitive to carry in ultra-care test performing procedures. Both testing procedures have their sampling, testing formats, and targets within the limitations to use. A comparative study reveals RT-PCR 36.6% results, and 17.3% serological assay tested positive while 6.8% showed positive serological testing and negative RT-PCR results [112]. So, it is not a confirmed shot to hit. Nevertheless, uncertainty regarding serological assays is much higher than in other methods. Serological testing includes antigen-based and antibody-based detection.

#### 2.8.1. Antigen-Based Detection

The SARS-CoV-2 contains the spike protein (S-protein), nucleoprotein (N-protein), envelope protein (E-protein), and membrane protein (M-protein) [17]. The S-protein exhibits a heavily intact glycosylated bond [113] and is a maximum mutation region, which may affect the effectiveness of the immunoassays. The N-protein is abundant to contribute to the identification of virus particles and RNA packages [114]. The antibodies IgA, IgG, and IgM against the N-antigen have an optimal expression in corona patient serum to run Immunoblast assays. Previously, N-antigen is taken as an impeccable detection marker for the prognosis of SARS-CoV and MERS [115], while these have a more genomic expression of N-antigen than SARS-CoV-2. The SARS-CoV-2 protein detection by the mass spectrometer method identifies samples collected from the gargles of the patients containing the viral load of 105 to 106 genome equivalents/μL, which is much smaller than the RT-PCR viral load requirement, suggesting its significance as an efficient diagnostic tool [116]. Comparing and selecting the more sensitive and specific recombinant antigens is essential before designing the serological assays. The S-antigen is involved in viral neutralization due to the domain (S1, RBD, and S2); as a result, it generates protective neutralizing antibodies, which can be commonly immobilized to develop assays [117]. The S-antigen-based assays’ sensitivity and specificity are higher than those of the N-antigen and have a lower cross-reactivity [118]. In a study, a quantum dot-based lateral flow immunoassay was found to be more sensitive for the detection of N-antigen than a particular conventional ELISA with a relevant recorded LOD of 100 ng/mL and 10 ng/mL [119]. Furthermore, an immunochromatographic fluorescence assay was designed to detect N-antigen from the patients’ nasopharyngeal swabs and urine samples in 10 min. The urine samples of corona patients were detected with 73.6% (14/19) N-antigen; overall, a 100% positive results accuracy was shown even after the negative nucleic acid test results of the same samples [120], while the urine sample of SARS recorded 8% [121]. The possibility of low viral protein expression in body fluids of SARS-CoV-2 patients verifies the data on a more significant number of samples [122]. It is more convenient to test the N-antigen of SARS-CoV-2 than other antigens because it is abundantly expressed in serum, although both antigens have greater immunogenicity to the viral proteins.

#### 2.8.2. Antibody-Based Detection

A lateral flow immunoassay was designed to target the serum antibodies of (IgG/IgM) receptor-binding domain (RBD) of the spike protein of SARS-CoV-2. This sensor enabled the simultaneous screening of IgM and IgG from the serum of the patient with a precision of 90.63% and sensitivity of 88.66%. Samples from a fingerstick, plasma of venous blood, and serum gave consistently positive results in less than 30 min [120]. An IgM ELISA against N-protein was designed for the carrier’s humoral response to the infection from the onset of the symptoms until the patient recovered. The recorded positive detection rate between the sets of 0–7 days, 8–14 days, and 14–21 days from 208 plasma samples was 188/208 (90.4%) for IgM and 194/208 (93.3%) for IgA against the target. Comparatively, the IgG detection rate was 162/208 (77.9%) within seven days. Gradually, the rates stopped increasing within 14 days for both (markers of acute infection), while IgG kept increasing after 21 days. Considerably, the detection of IgM and IgA identifies the current infection and IgA for the post-infection response to the body. IgM’s positive rate reached 93.1% (54/58) within the 5th day after the onset of the symptoms. Therefore, it is considered a competent testing method compared to the qPCR testing with an increased positive rate of qPCR [104,121]. Similarly, the response found for SARS-CoV in 2003 for IgM developed in 3–6 days, and IgG was detectable after eight days of onset of symptoms [123,124]. However, IgM gives the first line of defense against infection than IgG [125].

### 2.9. Challenges in Immunoassay-Based Diagnostics

Immunoassay is an efficient detection method yet has some uncertainties. Serological testing helps us to interpret the disease severity, ascertain the immunization level, identify updates against the past infection, and measure the vaccine efficacy. Each person before and after the onset of symptoms until the recovery has their immunity responses. A cascade of antibodies generation according to specific antigens is produced at the different periods within 0–7, 7–14, and 14–21 days after infection. The seroconversion-based detection depends upon the antigenicity of antigens. A similar trend of antibody generation was observed in the past infections such as SARS-CoV and MERS. Due to specific antibodies, IgM against RBD is much stronger compared to the IgM-N-protein depending upon the antigenicity. Because it usually takes up to 1–2 weeks for the human B-cells to produce and secrete antibodies in the blood serum after a viral infection and during the early stages of the COVID-19 pandemic, an optimal level of antibodies was not known [126]. The antigenicity of both antigens is highly noticeable, as SARS-CoV patients had higher anti-N than anti-S opposite to SARS-CoV-2, which has higher anti-S [nAbs of RBD] because of the highly conserved spike region. IgG rates against both antigens are higher than the IgM, while total seropositivity (IgG/IgM) against anti-S is higher than anti-N. In conclusion, S-antigen has higher antigenicity than N-antigen; while entering into the host cell, the viral (S1, RBD, and S2) domains become fused with the ACE 2, resulting in a quicker immunological response to develop protective neutralizing antibodies. The developed assays-based kits in Table 1 summarize the developed techniques for the detection of antigen, antibody, and combined antibody testing [127]. It would be critical to consider serological testing for the current infection as authentic as other methods. The availability of the targeted antigen, adequate antibody generation, and time and site to collect samples may cause the false diagnosis. Despite diagnostics apprehensions, immunoassay has remained the most reliable diagnostic after PCR testing.

## 3. Biosensors

The biosensors are analytical devices that consist of (i) a bioreceptor to recognize the analyte (biomolecules such as RNA, DNA, enzymes, cells, and aptamers), (ii) a transducer to turn the biorecognition into detectable signal readout, and (iii) a display to read the detected signal [128]. Biosensors exhibit a signal detection system with a minimum manual operation to reduce errors. To control the spread of the pandemic from asymptomatic and mildly symptomatic patients without leaving their places, a device for sample-to-result detection of SARS-CoV-2 is essential. In 2017, the WHO designed an affordable, sensitive, specific, user-friendly, rapid, robust and equipment-free deliverable device (REASSURED) for nucleic acid-based detection of HIV [129]. Similar efforts have been made for the development of devices according to targets such as immunosensors for antigen (N, S, and E-proteins) and antibody (IgG, IgM, IgA); modification of the critical step of RNA extraction to detect nucleic acid by using electrochemical biosensors, magnetic particles, and lab-on-chip microfluidics was adopted. Reusable, recyclable materials for the sustainability of the clinical diagnosis of masses are the requirement. This section discussed promising techniques for the development of biosensors for the detection of SARS-CoV-2 in clinical samples. Compared to conventional detection methods, biosensors are promising, innovative, reliable, rapid, sensitive, accurate, multiplex detection, and user and eco-friendly devices that are beneficial for the crowd testing. Table 2 summarizes the developed biosensors for the detection of SARS-CoV-2.

### 3.1. Biosensor for Genome Based Detection

A chip-based model was designed for the detection of extracted viral genome in less than 20 min. The followed principle of RT-LAMP was incorporated within the chip [145]. A nylon mesh medium was designed to use the rolling circular amplification technique to form DNA hydrogels. The nylon mesh surface overlapped onto a glass surface containing microholes, which were later blocked by hydrogels to control the flow in the attached tube. A maximum number of the target can be attached by rotating a magnetic bar due to the less effective area between the target and nylon mesh. The nylon mesh surface overlapped onto a glass surface containing microholes, which were later blocked by hydrogels to control the flow in the attached tube. A maximum number of the target can be attached by rotating the magnetic bar due to the less effective area between the target and nylon mesh. The nylon mesh area has been blocked by the DNA hydrogel infusion into micro-holes. The microfluidic system enabled the detection of SARS-CoV-2 up to the lowest concentration of 3 aM in 15 min and 30 aM in 5 min [146]. Likewise, a microfluidic system based on the CRISPR technique was developed for monitoring the viral genome from clinical samples taken from nasopharyngeal swabs. An ITP-based chip was obtained by applying an electric field gradient onto a microfluidic chip through an ion focusing technique, which is a different approach than typical CRISPR-Cas-12. The same ITP provided an automated platform for the purification of the target RNA with loop-mediated isothermal amplification and ITP-modified CRISPR enzymatic reaction for the detection of SARS-CoV-2 RNA within 35 min. The assay run by the reagents is less than 0.2 μL, which is 100 times less than the existing CRISPR method [147]. A plasmonic biosensor functioned to perform the reliable, rapid, and sensitive detection of SARS-CoV-2 followed by two simultaneous principles of plasmonic photothermal (PPT) and localized surface plasmon resonance (LSPR) on a AuNIs chip. Complementary sequences of DNA receptors are immobilized on AuNIs surfaces and were hybridized to specific gene RdRp-COVID, ORF1-COVID, and E-genes from SARS-CoV-2 [131]. The AuNI chip generates thermoplasmonic heat when illuminated. Exciting LSPR and PPT at two different angles of incidence enhance the temperature to discriminate the hybridization kinetics. The in situ hybridization kinetics are enhanced because of plasmonic photothermal heat to differentiate the sequences of RdRp of SARS-CoV and SARS-CoV-2. Therefore, it provided a more specific detection platform due to its unique feature to discriminate the coronaviruses.

### 3.2. Biosensors for Surface Protein of SARS-CoV-2

#### 3.2.1. Lateral Flow Immunoassays

**Figure 4:**
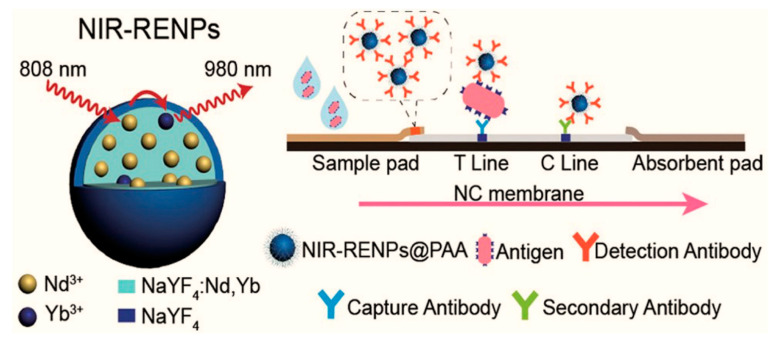
Near-Infrared Lanthanide—Doped Nanoparticles (NIR-RENPs), lateral flow immunoassay test format for the viral antigen detection. Reproduced with permission [124]. Copyright © 2020 American Chemical Society.

The lateral flow immunoassays (LFIAs) are easy-to-use, inexpensive, sensitive, and reproducible for the detection of SARS-CoV-2, and they have been also used for the detection of RNA [74,148]; still, to enhance the sensitivity, nanomaterials such as quantum dots and magnetic nanoparticles have been applied as immunolabels [149]. The nanomaterials with longer lifetimes periods of florescence have less background noise, and time-resolved analysis increases the sensitivity of the assay [150]. For the commercial application of LFAs, a low limit of detection of assays was achieved by using lanthanide-doped nanoparticles with a near-infrared (NIR) wavelength emission range (Figure 4) [124]. This platform showed high sensitivity without any interference.

#### 3.2.2. Lateral Flow Assays

Lateral flow assay (cassettes or strips) is the most prominent rapid diagnostic tool in POCT such as pregnancy strips [151]. These have been applied in several other fields for diagnostic purposes. Lateral flow assays (LFAs) are formatted on a viral RNA, antigen, or antibody basis for the detection of SARS-CoV-2. Generally, the test strip is based on a sample pad, conjugating pad and absorbent pad containing the Test line (T-line) and Control line (C-line) on a nitrocellulose base. Recently, LFAs were upgraded by applying RT-LAMP [152,153], CRISPR/Cas-9 [154], and RT-PCR based for the viral RNA detection [155,156]. Similar efforts are made for the detection of IgG, IgM-based LFAs [157,158] and antigen-based testing [159]. Furthermore, aptamer-based LFAs are being taken on account due to high sensitivity, long shelf life and cost effectiveness [160,161,162]. Based on the available genomic data, the LFAs can be used to design the newly mutant strains of SARS-CoV-2. LFAs are applied for the diagnosis of current infection. Beyond the rapidness and cost effectiveness, it has the limitation of identifying only the past infection.

#### 3.2.3. Microfluidic-Based Immunosensor

The magneto immunosensor was developed for the detection of the N-protein from the whole serum of 50 pg/mL and a diluted sample of 10 pg/mL. The sensitivity increased up to 230 pg/mL for concentrated and 100 pg/mL for diluted samples after integration of the microfluidic chip with a smartphone. The sensing platform was developed by incorporating dually labeled magnetic beads for immunogenic enrichment and the amplification of signals on a microfluidic chip [163]. Such hand-handled devices support less dependency on heavy instrumentation and enable the rapid monitoring of SARS-CoV-2 in bio-fluids.

#### 3.2.4. FET-Based Biosensor

An electrical biosensor detects the analytes and small molecules after a change in surface potential due to the binding of the target to the detecting species. The target was immobilized onto the surface of conducting channels made up of semiconducting materials such as graphene, molybdenum disulfide, zinc oxide, and gallium nitride. The FET device utilizes the surface potential gradient to generate a readable small amount of signal after detection [164,165]. Previously, FET devices have been used for the quantitative real-time detection of the Ebola virus. Recently, a FET-based sensor was designed for the detection of S-protein of SARS-CoV-2. The platform achieved the LOD of 1 fg/mL. The sensitivity of signal production is mainly because of the used conducting material. Among other 2D materials, graphene is considered reliable to sense a small change. It possesses high conductivity due to different electronic spectrums, low-dimensional flexibility, and electron charge mobility. Therefore, graphene-based FET is the best to choose for immunological detection [166,167].

### 3.3. Biosensors for the Detection of Antibodies

Microfluidic chip-based devices enabled us to perform the serological testing of COVID-19 from the blood plasma (1 μL diluted in 1 mL buffer solution) of the patient by the limit of detection of 0.08 ng/mL (0.5 pM), which is in the range within the clinical concentrations of antibody (IgG) against S-protein. Gold nanospikes were fabricated through electrical deposition using the localized surface plasmon resonance (LSPR) technique. A shift in the wavelength peak of gold nanospikes was measured as a change in the refractive index after antigen–antibody binding. Yet, this method needs to be optimized for multiplex testing and validated for other targets in the blood before commercial application [144]. A simple multiple target on-site detection of SARS-CoV-2 on a microfluidic system was proposed recently. The detection of antibodies IgG and IgM was achieved in less than 15 min from the blood, plasma, serum, and alveolar fluid samples [168]. However, the monitoring of other respiratory pathogens is required to evaluate the specificity of the system. More innovation is brought to immunoassay techniques during this pandemic. The simultaneous detection of IgG/IgM and antigen detailed the current and past infections and confirmed the accurate diagnosis of the patients. Furthermore, the application of smartphones has facilitated the resulting interpretations [169]. Nano immunoassay was developed by designing 1024 sample units in a PDMS chip by following the pattern of a 1024-cell serum analyzer, and it was even close to the MITOMI device with a slight change in enlarging the size of the chambers to carry the presented samples [170]. A high-throughput microfluidic device was designed for the detection of anti-SARS-CoV-2 antibody IgG. Samples were loaded by a contact-printing microarray robot onto the epoxy-coated glass slide overlapped with a PDMS device [171]. This approach has the potential for the detection of the virus during a pandemic situation to test immunity development at the different stages of disease for thousands of patients simultaneously.

### 3.4. Miscellaneous Biosensor Technologies

#### 3.4.1. Multiplexed Paper-Based Colorimetric Sensor

Paper-based devices (PADs) are inexpensive, equipment-free, independent of trained staff portable, and user-friendly in point-of-care testing [172,173]. A colorimetric paper-based sensor was designed for the detection of MERS-CoV using AgNPs of pyrrolidinyl peptide nucleic acid (acpcPNA). Upon the interaction of acpcPNA and complementary DNA, the AgNPs dispersed due to electrostatic repulsion, which causes a visible color change on the paper strip [174]. An LOD of 1.53 nmol/L was observed, and the procedure can be easily applied for the detection of the viral genome of SARS-CoV-2. Conventional labeled (AuNPs) LFAs have low sensitivity and multi-user steps, which may cause an imprecision of application. Further modified enzyme-based signal amplification also increases the complexity of the assay. An ultrasensitive and financially viable LFA biosensing new automated immunoreaction and amplification scheme was designed. AuNPs were incorporated with antibodies into a polymer network peroxidase, and significantly programmed amplification on a hydrophilic polymer was used to detect target [175]. The commercialization of these devices is underway [176]. A low-cost technique of paper-based lateral flow immunoassays to detect infectious diseases is applied in several developing countries [177].

#### 3.4.2. Nanotechnology-Based Sensors

Nanoengineering plays a promising contribution in various disciplines, including agriculture, environmental sensing, and clinical diagnosis and treatment [178,179]. Viral surveillance is complicated, especially in crowded airports, hospitals, shopping malls, institutes, subways, and clubs. To screen positive carriers from a single drop of serum, plasma, or blood on the spot would be helpful to overcome the massive chaos after any future outbreak. Sensitive COVID-19 testing strips for bedside monitoring can be innovative. In addition, a satisfactory entrance of masses to the prominent places required installed rapid sensors for on-field detection. The successful extraction of the viral RNA was performed by using nanomaterials such as nanofibers, nanoparticles, and composite membranes [180]. A lateral flow immunoassay coupled with the colloidal gold particles on a nitrocellulose-coated surface (Colloidal gold immunochromatographic assay (GCIA)) was compared with the conventional immunoassay for the IgM and IgG. The sensitivity of combined ELISA IgG and IgM was 87.3% higher than the GCIA assay for combined IgG and IgM (82.4%), but the 100% specificity for the assays was the same. Such assays would easily be applicable for the detection of multiple targets [181]. Furthermore, promising nanomaterials, such as polysulfone ultrafiltration membranes containing silver nanoparticles, reported removing bacteriophages [182]. Nanomaterials to remove the viral genome can also be subjected as a nanomedicine in severe patients. The composites of polyacrylonitrile (PAN) with electrospun nanofibers membranes (ENMSs), nanofibers of ammonium tetrathiomolybdate (ATM), and tetraethoxysilane (TEOS) provide a rough surface to detect and remove bacteria and viruses from the samples within the efficacy up to ˃90% [183]. The nanoparticles were imbibed onto membranes to remove and screen the viruses from the samples for the naked-eye detection of N-antigen through a color change in 10 min [132]. A rapid and direct capturing of viral particles was achieved by using polymer tentacles-based functionalized magnetic latex. The magnetic particles coated with the captured antibody easily captured the virus directly from the surfaces and environment. Subsequently, RNA was extracted from the attached virus. This method can be applied generally for the detection of other pathogens as well. Nanoparticles-based lateral flow biosensors (LFB) are affordable to detect several analytes and biomolecules. Portable tools are utilized by coupling several other biosensing techniques such as RT-LAMP [152,184], and also, lateral flow immunoassays (LFIAs)-based biosensors are applied for the detection of SARS-CoV-2 [185,186].

#### 3.4.3. Aptamer-Based Detection

Aptamers have advantages over antibodies due to their high stability with a wide range of temperature and their method of synthesis (SELEX) and cost-effectiveness [187]. N-antigens of SARS-CoV were detected in blood serum using specifically designed aptamers 1 and aptamer 2, which bind on the specific C-terminal to detect the target gene by a low dissociation constant of 0.81 nmol/L and 3.35 nmol/L. Moreover, possessing a greater binding affinity than antibodies, this technique can be applied for the detection of SARS-CoV-2 [188]. The packing RNA nanoparticles (pRNA) were functionalized with aptamers, ribozymes, small interfering RNA, and microRNAs for the monitoring of viral particles [189]. Viral and genetic diseases in humans, animals, and plants can be diagnosed using similar techniques.

#### 3.4.4. Artificial Intelligence-Based Sensors

Artificial intelligence (AI) is among the most advanced applied techniques for detection, surveillance, and tracing the evolutionary history of COVID-19. In recent studies, it has been used to trace its evolutionary linkage with other viral families such as Coronaviridae, Flaviviridae, Retroviridae, Flaviviridae, and Orhomyoxyviridae due to high peaks sharing S, L, and T amino acids. Moreover, the AI-based technologies can help to trace mutations and predict future evolution [190,191].

#### 3.4.5. Electrochemical Biosensors

A biosensor was designed to detect MERS-CoV for simultaneous coronavirus detection and relied on the indirect competitive immunoassay. The gold nanoparticles containing free virus and MERS-CoV protein at a fixed concentration of added antibody was immobilized on a carbon electrode array. The sensor response was detected by monitoring the change in the peak current upon adding different concentrations of the MERS-CoV antigen [192]. Likewise, an impedimetric sensor was developed for the multiplex and label-free sensing of the influenza virus. A decrease in redox flux and an increase in impedance were observed after binding between antigen and antibody [193]. For the current needs, the development of electrochemical-based biosensors is a prototype, but for an ideal approach, their application might be restricted to a few samples under the lab compulsion. The sensitive temperature ranges, short shelf life, and low viral loads are challenges in developing such biosensors.

#### 3.4.6. Surface Plasmon Resonance-Based Biosensor

**Figure 5:**
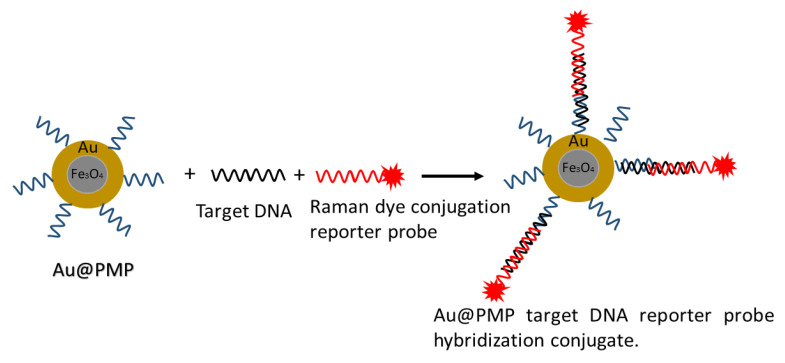
Antisense oligonucleotide for the direct detection of the viral genome from the gold nanoparticles, ASO labeled with Raman active compound for the high specificity. Reproduced with permission [198]. Copyright © 2012 American Chemical Society.

Surface plasmon resonance (SPR) is one of the excellent technologies to design sensors for a wide range of biomarkers. These devices can phenotypically map the binding events in a highly prudent and temperature-controlled automated system. The SPR-based devices can be developed to have multi-channels for simultaneous multiplex detection (Figure 5). The application of SPR-based surfaces for the evaluation of multiple samples can overcome the bio waste materials including multiple testing strips and kits used during analysis at home and in crowded places. SPR has been frequently applied for the detection of various respiratory pathogens. The ferromagnetic pattern on a substrate was achieved for the monitoring of the H1N1 influenza virus in a total run time of 1.5 h [194]. This run time was relatively higher than the assays designed for the detection of other viruses. An inhibition assay was designed on a semi fluidic sensor chip with immobilized HA protein for the quantification of two proteins H1N1 and H3N2 of AIV in 10 min [195]. A microarray pattern was obtained on the SPR biochip for the identification of the origin of assembly in a specific RNA fragment to evaluate the role of viral proteins [196]. Similarly, genetically fused gold binding polypeptides were combined with coronaviral surface antigen and immobilized on the surface of a chip for selective detection of the target [197].

#### 3.4.7. Localized Surface Plasmon Resonance

Localized surface plasmon resonance (LSPR) is created in nanospheres particles by an externally applied electromagnetic field. The plasmonic wavelength is dependent on shapes, scattering to absorption ratio, and extinction site. LSPR has been used to detect the surface antigens of several enveloped viruses such as hepatitis B virus, avian H5N1, Ebola, dengue, and currently for SARS-CoV-2 with the different strategies of antibodies–antigens immobilization to capture targets. Thiol-modified antisense oligonucleotides (ASOs) are used for the naked-eye detection of SARS-CoV-2. Furthermore, the close proximity between fluorophores and nanostructures enhanced the resonance effects [76,132,199,200]. The FRET can be observed if an optical absorption spectrum and electric field of sensing material overlap. The metal’s electric field has a larger absorption spectrum for the LSPR plasmon for a highly efficient FRET. Therefore, a fluorescence immunoassay-based nano biosensor was designed for the detection of the influenza virus [201]. This approach can be also applied to the detection of SARS-CoV-2.

**Surface-Enhanced Fluorescence (SEF)** biosensors have been successfully used to detect HIV serotypes on polystyrene substrates. Immobilized antibodies were captured from a complete blood sample using the nanoplasmic property of AuNPs, with a limit of detection of 39 copies/mL for serotype D. [202]. A variety of fabrication strategies can be subjected to immobilize the antibodies, such as metallic nanoparticles (Ag@SiO_2_ NPs, multi plasmonic gold) conjugated with the linkers to detect several other viruses on the same lateral flow platform. The applicability of this not only improves the total assay run time but also increases detection limits, requires fewer sensitive steps, and enables direct detection of the target from the sample with more specificity. Nanospheres coated with the antibody of the Ebola virus immobilized on the test line in a lateral flow immunoassay demonstrated the excellent application of SEF-based devices [203,204].

**Surface-Enhanced Raman Spectroscopy (SERS)** A series of studies are conducted to detect enveloped viruses, adenovirus, rhinovirus, human immunodeficiency [205], respiratory syncytial virus [206], and rotavirus [207] using the SERS technique. Multilayered arrays of nanorods of Ag-Au served as the SERS substrate for detecting influenza virus strains H1N1, H2N2, and H3N2 at 106 pfu/mL concentration [208]. The plaque-forming unit (pfu/mL) measures the infectivity of the viral suspension [209]. The rapid detection by coining lateral flow assay strips and photonic-based technology are implemented before for influenza and respiratory viruses. Lateral flow strips were also integrated with SERS to detect nucleoprotein of influenza virus A by conjugating nanoprobes (multibranched AuNPs) with the Raman reporter [210,211,212].

#### 3.4.8. Biosensors Designed for Alternative Target of SARS-CoV-2

M^pro^ protease is critical in the viral replication of SARS-CoV-2 and is an alternative target to quantify the infection rate and progression of infection [213]. The challenges include estimation of the incubation period, progression period, low expression of viral load, and presenting as asymptomatic, but the carriers can be addressed by targeting viral replication proteins. The expression of these replicating proteins can quantify directly in the serum or blood of the patients. Until recently, fundamental experimental approaches have mostly been used on RNA, surface antigens, antibodies, and viral particles. The tests are intended to target anti-SARS-CoV-2 drugs. Mpro, RdRp, and PLpro are proteins involved in viral genome transcription and replication that also serve as detection targets. Any structural protein, including E, N, S, and M proteins, can be translated using negative chain RNA as a template [214]. Several biosensors and tests have been designed using replication proteins other than structural proteins. The RNA extraction-free detection assay was found to be significantly less expensive and simple to apply for the detection of SARS-CoV-2 [215]. When compared to proteins involved in viral entrance, infusion, and replication, conserved proteins are more suited for detection. Rather, these are ideal for developing effective medications such as Mpro and PLpro. The quantification of infected cells through plaque assays is applied to measure the infection entity of the viruses [216], but it is limited to high-throughput application. An assay was designed in which viral protease expression is utilized to quantify infected cells. The cleaved oligopeptide linkers activated cell-based optical biosensors. Furthermore, the recombinant viral proteases measures the capacity to detect SARS-CoV-2 protease expression during actual virus replication in infected cells. The reported cell line based on luciferase biosensors quantified the viral infection within 24 h [217].

## 4. An Overview of the Commercially Available Kits

The data from the FIND pipeline were analyzed to overview the molecular assay kits, immunoassay kits, and kits based on other detection strategies.

**Figure 6:**
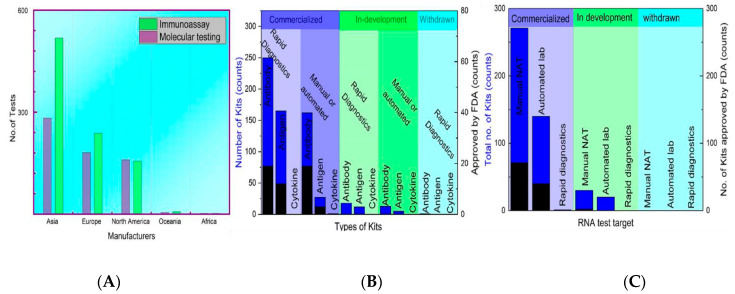
FDA-approved commercial kits: (**A**) The number of testing kits (molecular and Immunoassay based) produced by the Global manufacturers (**B**) Status of molecular-assay based kits, (**C**) Status of serological assay based.

These data describe globally accepted commercial kits with a precise result interpretation—the assays that are still in development. Based on rapid and manual/automated diagnosis, the FDA-approved technologies are summarized in Figure 6. Molecular assay and immunological assay-based home testing, pool testing, and sample collection kits have seen massive production globally. Moreover, currently, as of September 2021, the FDA approved the total collection of 235 molecular, 88 antibodies, and 34 antigen tests among 400 total tests, including 63 home collection kits, 32 pooling testing kits, 55 point-of-care, 19 multi-analyte, and 13 at-home on a shelf collected samples [218].

The high cost of NAAT testing limits its commercial application in low-resource localities [219]. At the same time, supplementary testing is inexpensive and fast [220]. The LFIA using latex microspheres is fast, user-friendly, and highly cost-effective; it is only $0.15 per test [221]. However, the production cost of the techniques varies according to available medical resources. The minimum commercial cost of the available kits is recorded at $29.63, and the highest is $150 and $140 for antibody testing [222]. In Figure 6A, according to the FIND database, 1570 total testing kits have been designed, out of which 639 are molecular assays-based and 925 are immunoassay-based testing. The production cost of the techniques varies, but manufacturers are providing the low commercial prices of testing kits from all over the world.

## 5. Impacts of Mutants on Diagnosis

The intervention of the mutants was observed when in many countries, the constant false-negative results were obtained for the hospitalized patients. It draws attention to the detailed analysis of the sequencing data. Mutations with biological impacts detected in SARS-CoV-2 cause the immune escape to the possibility of reinfection within a short span. Table 3 presents the detected VOCs and VOIs and their potential clinical changes in antigenicity, virulence, and transmissibility. Mutations are the changes that occurred in the peptide sequence—addition, deletion, or a nucleotide substitution—and these may cause harmful, advantageous, or no effects on the remaining sequence. For SARS-CoV-2, it is seen that the mutations in the peptide sequence of S-protein with 1273 amino acids have a key role to induce viral entry and viral binding at the receptor-binding site of the host cell. It also increases transmissibility, but on the other hand, the antigenicity, virulence, and vaccine escape of the variants may become more affected if the mutations occurred on the N-gene or at the viral genome to induce phenotypic changes.

The occurrence of escape mutation indicates the effects on immunogenicity and the recurrence of the infection. Furthermore, experiments and data can interpret the actual effects. Table 3 contains the automated naming of genomes that were added by using online software for the naming and information of active lineages of SARS-CoV-2 variants along with their mutations [235,236,237,238]. The false testing results of SARS-CoV-2 could be challenging, especially for those who rely on only single target detection, but for multiple target detection, it may be less risky. The variants have an impact on antigen and molecular-based analysis due to the specific mutation regions on the S-antigen. For PCR testing methods, a set of primers may not bind to the target binding site due to the change in the peptide sequence. Simultaneously, the false antigen testing is due to the absence of specific epitopes for the detection antibody. In many regions, false antigen testing has been used as a proxy to track B.1.1.7 variants, but it has low reliability compared with sequencing-based analysis.

The number of mutations in the spike regions of the virus is found to be more than in the non-spike regions. It increases the transmissibility and virulence of the pathogen. The variants B.1.1.7 [239,240], B.1.351 [228,237,241], B.1.617.2 [230], B.1.525 [242,243], P.1 [244], and A.23.1 [245] have significant mutations at the different sites.

## 6. Summary and Future Directions

The detection of SARS-CoV-2 is being challenged by some unmet problems, which need to be resolved by some innovative solutions. The SARS-CoV-2 RNA detection has been significantly modified for the sensitive step of RNA extraction. The dependency on centrifugal steps, organic solvents and temperature ranges (mostly extractions are performed at room temperature) increases the sensitivity to handle samples taken for the detection [246,247]. Moreover, a longer time is needed for nucleic acid isolation, which is unfavorable for rapid diagnosis. Several methods such as BSA-based protocol [248], heat shock-based [249] and acid pH method [250,251] are taken to conduct the RNA isolations of SARS-CoV-2. The low viral loads hinder the accurate screening of the viral genome that cause false-negative testing [252,253,254,255]. Stable viral lysis buffer at different temperature ranges [256], acid pH ranges [257], and RNA extraction free detection [248,251,257,258,259] are the promising strategies for viral RNA storage and stability. Furthermore, the RNA stabilization method for a long duration at room temperature can meet the limitations. Visual plasmonic sensors can be applied and integrated with several other strategies for naked eye detection [260]. Biosensors-based devices to detect SARS-CoV-2 and other viruses are the cornerstone of the multiplex testing system. Biosensors based on nanotechnology, microfluidic chips, lateral-flow assays, paper-based devices, and others have been developed for sensitive, selective, simple, and fast detection in homes and crowded places. The best potential methods for providing the direct early indication of SARS-CoV-2 infection are SARS-CoV-2 NP and virus particle detection. The application of viral replicating protein as a detection target is a next step toward the diagnosis of new variants also. The testing schemes based on the single target detection may not work in the future. Automated SPR-based biosensors could be reliable, sustainable, and precise monitoring devices without extensive dependency on trained staff and lab safety. Serological tests are significant for the monitoring of past infection, vaccine efficacy, immunity development concerning disease severity, and viral antigenicity. Even after vaccination, serological testing can interpret the antibody level to boost immunity.

The majority of the technologies are used to target S-proteins, antibodies, and N- and S-antigens. However, solutions such as the on-site diagnosis of asymptomatic COVID-19 patients with minimal time lag would be exceptional. This challenge might be solved by combining single-step nucleic acid amplification with a noise-resistant PCR process integrated on a chip to save time, eliminate labor reliance, and provide low-cost detection. There is also the monitoring of antigen-related problems found in infected individuals, such as a mutation in the target antigen area [255] with N-antigen transcriptional expression being higher in cells infected with coronaviruses other than SARS-CoV-2 [122]. Point-of-care testing equipment for multiplex detection or tests that detect trace levels of antibodies might do this. Furthermore, the ID NOW COVID-19 technique can detect viral genomes in minutes, which is a cutting-edge application. However, the bio waste generated by the usage of swabs and sample collecting tubes remains a challenge. For multiplex detection employing sensing platforms, a fixed, cleanable, and reusable cartridge on the device without disposable testing strips and a fixed signal reader from a single swab rub or direct sample drop is required. Machine learning algorithms might eventually replace PCR testing. Similarly, a machine learning-based relative synonymous codon use frequency (RSCU system) can quickly decode viral sequences from unknown strains. Because of technology support systems for survival in this biosphere, biodiversity, climate, and immunity are being compromised. Before transforming our global home into a technosphere, engineers and ecologists should work together to eliminate bio waste hazards [261]. Individually, COVID-19 posed two key challenges: isolation and immunity. Vaccination is presently the only effective therapeutic option. Furthermore, confinement in our homes or hospitals generates stress and anxiety in many people, similar to what astronauts endure during long-term space missions [262]. After adjustments to the health monitoring technologies used for astronauts, such as 3D printing instruments and stress management countermeasures, the terrestrial lifestyle might benefit to counteract any future epidemic.

## Figures and Tables

**Table 1:** Diagnostic Methods of SARS-CoV-2.

**Molecular Assay**	**RNA**	**Test Format**	**Turnaround** **Time (min.)**	**Target Analyte**	**Validated Samples**
		NAT reagent kit (Open source)	60–120 <13 [32] 40–50 [33] 83 [34] 8 h (188 samples) [35]	N-gene,E-gene, S-gene, ORF1ab, RdRp gene, ORF1a,	Nasopharyngeal swab, Unknown, Oropharyngeal swab Nasal swab Bronchoalveolar lavage
		Cartridge based qRT-PCRRT-LAMP	36 [36], 45 [37], 30 [38]	Orf1ab, N-gene, RdRp	Nasopharyngeal, Oropharyngeal, Nasal swabs
		Dipstick CRISPR-Cas-12	<40 [39]	Orf1ab, N-gene	Nasopharyngeal
**Immunoassay**	**Antibody(serological)**	Cartridge-based processing	15 [40]	IgG, Total Antibody, IgM	Serum, Plasma
		Chemiluminescence	15 [41] 29, 80, 120, 48 [42]	IgG, IgM, Total Antibody, Nucleocapsid protein, Unknown	Serum, Unknown samples
		Rapid diagnostic Lateral Flow ELISA Colorimetric	15–20 60–90 [43] 10 [44]	IgG,N-protein, Total antibody Unknown	Serum Plasma Unknown saliva
		Reagent Kit LFIA ELISA	20 [45] <90 [46]	IgG, Total antibody,IgA,N-protein, Unknown	Serum Nasopharyngeal Unknown
	**Antigen**	Cartridge-based process Chemiluminescent immunoassay (CLIA)	15 [47]	N-protein, S-protein (RBD) N-protein	Nasopharyngeal swab Saliva Nasal swab Unknown Oropharyngeal swab
		Rapid diagnostic Strips Cassettes	15 [48] 20–30 [49,50]	N-protein S-protein (unknown type) S-protein RBD S-protein S1 S-protein S2	Nasopharyngeal swabs Nasal swab Saliva Unknown Oropharyngeal swab Sputum
		Reagent kit	15 [51]	N-protein S-protein S1 S-protein (unknown type)	Nasopharyngeal swab Unknown Serum Nasal swab Oropharyngeal swab

**Table 2:** Developed biosensors for the detection of SARS-CoV-2.

**Target**	**Biosensors**	**Principle**	**LOD/Detection Time/Cutoff Value/Sensitivity, Specificity**	**Drawbacks/Targets**	**References**
**Genome Based Detection**	Label-free electrochemical biosensor	DNA hybridization of electrodeposited AuNPs immobilized with single-stranded nucleotide	-	RNA extraction Sensitive sample handling	[130]
	Plasmonic biosensors (optical-LSPR)	Dual-functional plasmonic biosensor combining the plasmonic photothermal (PPT) effect and localized surface plasmon resonance (LSPR) sensing transduction	0.22 pmol/L, 3 min	From multigene mixture	[131]
	Naked-eye colorimetric	Thiol-modified antisense oligonucleotide is used to cap AuNPs, which change color upon finding the target N-gene	0.18 ng/μL, 10 min	N-gene	[132]
**Viral Particle Based Detection**	Cell-based biosensor	Membrane-engineered mammalian cell containing antibody to bind with S-antigen	1 fg/mL, 3 min	Not applicable for the detection of variants.	[133]
	Nanoplasmonic sensors	Optical measurement of the SARS-CoV-2 particle	370 vp/mL, 15 min	Restricted for the S-antigen	[134]
	Field effect-based transistor	Graphene-coated sheets with SARS-CoV-2 antibody	2.42 × 10^2^ copies/mL (in clinical samples)	s-antigen	[135]
	G-druplex-based biosensors			Whole genome	[136]
	Molecularly imprinted polymers	Monoclonal-type, synthetic antibodies of SARS-CoV-2	-	Only applied to the S-antigen	[137]
	eCovSens	Potentiostat-based sensor fluorine doped tin oxide + AuNPs immobilized with monoclonal antibody	90 fmol/L, 10–30 s	S-antigen	[138]
	Electrochemical Biosensor	Functionalized TiO_2_ nanotube-based electrochemical	0.7 nmol/L, 30 s	S-glycoprotein	[139]
	Lateral flow immunoassay	ACE2 enzyme binding captured antibody	1.86 × 10^5^ copies/mL	Spike antigen	[140]
**Antibody Based Detection**	Lateral flow immunoassay	Lanthanide-doped Nanoparticles	0.06666, 10 min	Anti-SARS-CoV-2-IgG	[141]
		Immunochromatographic	15 min, 85.2% and 100%	IgG-IgM combined	[142]
		Immobilization on AuNPs	15 min, 88.66% and 90.63%	IgG-IgM combined	[40]
	Plasmon-enhanced biosensor	Grating Coupled Fluorescent Plasmonic (GC-FP) based on ELISA from dried blood spot samples	30 min 100% and 100%	Multiplexed (IgG, IgM, IgA)	[143]
	Opto-microfluidic	A microfluidic device fabricated by the electrodeposition of Au-nanospikes linked with the optic probe to detect the target by using localized surface plasmon resonance	0.5 pmol/L, 30 min	Antibodies against the spike protein	[144]

**Table 3:** Variants of SARS-CoV-2 and the significant mutations at the Spike protein.

**Variants (Notations, Lineages)**	**Identification Date and Countries and Total No. of Countries**		**Sequences (GISAID)**	**Notable Mutations Occur at S-Protein Notable Mutations (S-Protein)**			**Effect on Antigenicity**
**(Alpha)** **B.1.1.7(VOC-202012/01,** **20/501Y.V1)**	UK, 20 November 2020	168	1,133,025	23	8	N501Y, [223] D614G [224], P681H [225]	N501Y effects on RBD [226] No effect on the serum neutralization [227]
**(Beta)** **B.1.351, 501Y.V2;** **20C/501Y.V2**	South Africa, 20 December	110	10,095,100	21	9	K417N, E484K, N501Y, D614G, A701V	K417T possibility escaping some monoclonal antibodies [228,229]
**(Delta)** **B.1.617.2**	India	141	10,095,100	12		S:P681R S:L452R,	Yes, Increased [230,231]
**(Eta)** **B.1.525**	Nigeria	80	9719	10		aa:S:E484K aa:S:Q677H aa:S:F888L	Yes, reduce serum neutralization against IgG [232].
**(Gamma)** **P.1, 501Y.V3,**	Brazil and Japan, 20 December	74	68,754	17	10	aa:S:E484K aa:S:N501Y aa:S:K417T aa:S:D614G, aa:S:H655Y	aa:S:E484K escape mutation affects the nAbs [233].
**A.23.1** **E484K**	Ugenda Brazil UK	48	1126	16	4	aa:S:F157L aa:S:V367F aa:S:Q613H aa:S:P681R	Yes [234]

## Data Availability

Not applicable.

## References

[1] Peiris J.S.M., Greenwood D., Barer M., Slack R., Irving W. (2012). Coronaviruses. Medical Microbiology (Eighteenth Edition).

[2] Su S., Wong G., Shi W., Liu J., Lai A.C.K., Zhou J., Liu W., Bi Y., Gao G.F. (2016). Epidemiology, Genetic Recombination, and Pathogenesis of Coronaviruses. Trends Microbiol..

[3] De Wit E., Van Doremalen N., Falzarano D., Munster V.J., Singh P., Hanson P.S., Morris C.M. (2016). SARS and MERS: Recent Insights into Emerging Coronaviruses. Nat. Rev. Microbiol..

[4] Azhar E.I., El-Kafrawy S.A., Farraj S.A., Hassan A.M., Al-Saeed M.S., Hashem A.M., Madani T.A. (2014). Evidence for Camel-to-Human Transmission of MERS Coronavirus. N. Engl. J. Med..

[5] Guan Y., Zheng B.J., He Y.Q., Liu X.L., Zhuang Z.X., Cheung C.L., Luo S.W., Li P.H., Zhang L.J., Guan Y.J. (2003). Isolation and Characterization of Viruses Related to the SARS Coronavirus from Animals in Southern China. Science.

[6] van der Hoek L., Pyrc K., Jebbink M.F., Vermeulen-Oost W., Berkhout R.J.M., Wolthers K.C., Wertheim-van Dillen P.M.E., Kaandorp J., Spaargaren J., Berkhout B. (2004). Identification of a New Human Coronavirus. Nat. Med..

[7] Wu F., Zhao S., Yu B., Chen Y.-M., Wang W., Song Z.-G., Hu Y., Tao Z.-W., Tian J.-H., Pei Y.-Y. (2020). A New Coronavirus Associated with Human Respiratory Disease in China. Nature.

[8] Neuman B.W., Adair B.D., Yoshioka C., Quispe J.D., Orca G., Kuhn P., Milligan R.A., Yeager M., Buchmeier M.J. (2006). Supramolecular Architecture of Severe Acute Respiratory Syndrome Coronavirus Revealed by Electron Cryomicroscopy. J. Virol..

[9] Woo P.C.Y., Lau S.K.P., Chu C., Chan K., Tsoi H., Huang Y., Wong B.H.L., Poon R.W.S., Cai J.J., Luk W. (2005). Characterization and Complete Genome Sequence of a Novel Coronavirus, Coronavirus HKU1, from Patients with Pneumonia. J. Virol..

[10] Andersen K.G., Rambaut A., Lipkin W.I., Holmes E.C., Garry R.F. (2020). The Proximal Origin of SARS-CoV-2. Nat. Med..

[11] Letko M., Marzi A., Munster V. (2020). Functional Assessment of Cell Entry and Receptor Usage for SARS-CoV-2 and Other Lineage B Betacoronaviruses. Nat. Microbiol..

[12] Chen Y., Liu Q., Guo D. (2020). Emerging Coronaviruses: Genome Structure, Replication, and Pathogenesis. J. Med. Virol..

[13] Lu R., Zhao X., Li J., Niu P., Yang B., Wu H., Wang W., Song H., Huang B., Zhu N. (2020). Genomic Characterisation and Epidemiology of 2019 Novel Coronavirus: Implications for Virus Origins and Receptor Binding. Lancet.

[14] Li F. (2016). Structure, Function, and Evolution of Coronavirus Spike Proteins. Annu. Rev. Virol..

[15] Chan J.F.-W.W., Yuan S., Kok K.-H.H., To K.K.-W.W., Chu H., Yang J., Xing F., Liu J., Yip C.C.-Y.Y., Poon R.W.-S.S. (2020). A Familial Cluster of Pneumonia Associated with the 2019 Novel Coronavirus Indicating Person-to-Person Transmission: A Study of a Family Cluster. Lancet.

[16] Hoffmann M., Kleine-Weber H., Schroeder S., Krüger N., Herrler T., Erichsen S., Schiergens T.S., Herrler G., Wu N.-H.H., Nitsche A. (2020). SARS-CoV-2 Cell Entry Depends on ACE2 and TMPRSS2 and Is Blocked by a Clinically Proven Protease Inhibitor. Cell.

[17] Schoeman D., Fielding B.C. (2019). Coronavirus Envelope Protein: Current Knowledge. Virol. J..

[18] Rota P.A., Oberste M.S., Monroe S.S., Nix W.A., Campagnoli R., Icenogle J.P., Peñaranda S., Bankamp B., Maher K., Chen M. (2003). Characterization of a Novel Coronavirus Associated with Severe Acute Respiratory Syndrome. Science.

[19] Weekly Epidemiological Update on COVID-19–21 September 2021. https://www.who.int/publications/m/item/weekly-epidemiological-update-on-covid-19---21-september-2021.

[20] Barber A., Griffin J., Casey M., Collins Á.B., Lane E.A., Ten Bosch Q., De Jong M., Evoy D.M., Byrne A.W., McAloon C.G. (2020). The Basic Reproduction Number of SARS-CoV-2: A Scoping Review of Available Evidence. medRxiv.

[21] Petersen E., Koopmans M., Go U., Hamer D.H., Petrosillo N., Castelli F., Storgaard M., Al Khalili S., Simonsen L. (2020). Comparing SARS-CoV-2 with SARS-CoV and Influenza Pandemics. Lancet Infect. Dis..

[22] Lv H., Wu N.C., Tsang O.T.-Y., Yuan M., Perera R.A.P.M., Leung W.S., So R.T.Y., Chun Chan J.M., Yip G.K., Hong Chik T.S. (2020). Cross-Reactive Antibody Response between SARS-CoV-2 and SARS-CoV Infections.

[23] Perlman S., Netland J. (2009). Coronaviruses Post-SARS: Update on Replication and Pathogenesis. Nat. Rev. Microbiol..

[24] Fehr A.R., Perlman S., Maier H.J., Bickerton E., Britton P. (2015). Coronaviruses: An Overview of Their Replication and Pathogenesis. Coronaviruses: Methods and Protocols.

[25] Coronavirus Incubation Period: How Long and When Most Contagious. https://www.webmd.com/lung/coronavirus-incubation-period#1.

[26] Zhou P., Yang X.-L., Wang X.-G., Hu B., Zhang L., Zhang W., Si H.-R., Zhu Y., Li B., Huang C.-L. (2020). A Pneumonia Outbreak Associated with a New Coronavirus of Probable Bat Origin. Nature.

[27] Shen Z., Xiao Y., Kang L., Ma W., Shi L., Zhang L., Zhou Z., Yang J., Zhong J., Yang D. (2020). Genomic Diversity of Severe Acute Respiratory Syndrome–Coronavirus 2 in Patients With Coronavirus Disease 2019. Clin. Infect. Dis..

[28] Wu J.-L., Tseng W.-P., Lin C.-H., Lee T.-F., Chung M.-Y., Huang C.-H., Chen S.-Y., Hsueh P.-R., Chen S.-C. (2020). Four Point-of-Care Lateral Flow Immunoassays for Diagnosis of COVID-19 and for Assessing Dynamics of Antibody Responses to SARS-CoV-2. J. Infect..

[29] MacMullan M.A., Ibrayeva A., Trettner K., Deming L., Das S., Tran F., Moreno J.R., Casian J.G., Chellamuthu P., Kraft J. (2020). ELISA Detection of SARS-CoV-2 Antibodies in Saliva. Sci. Rep..

[30] Mason M.G., Botella J.R. (2020). Rapid (30-Second), Equipment-Free Purification of Nucleic Acids Using Easy-to-Make Dipsticks. Nat. Protoc..

[31] Zhen W., Smith E., Manji R., Schron D., Berry G.J., McAdam A.J. (2020). Clinical Evaluation of Three Sample-to-Answer Platforms for Detection of SARS-CoV-2. J. Clin. Microbiol..

[32] Abbott RealTime SARS-CoV-2 Assay (EUA), Abbott Molecular. https://www.molecular.abbott/us/en/products/infectious-disease/RealTime-SARS-CoV-2-Assay.

[33] Altona DIAGNOSTICS RealStar ® Instructions for Use RealStar ® SARS-CoV-2 RT-PCR Kit 1.0 03/2020 EN. https://altona-diagnostics.com/en/products/reagents-140/reagents/realstar-real-time-pcr-reagents/realstar-sars-cov-2-rt-pcr-kit-ruo.html.

[34] CareStart COVID-19 MDx RT-PCR – Access Bio. https://accessbiodiagnostics.net/covid-19-mdx-rt-pcr/.

[35] SARS-CoV-2 Assay Kit | Applied BioCode. https://www.apbiocode.com/sars-cov-2.htm.

[36] Cepheid | Xpert® Xpress SARS-CoV-2 - FDA Emergency Use Authorization. https://www.cepheid.com/en/coronavirus.

[37] Respiratory Pathogen Panel Test | BioFire Diagnostics. https://www.biofiredx.com/products/the-filmarray-panels/filmarrayrp/.

[38] Amaral C., Antunes W., Moe E., Duarte A.G., Lima L.M.P., Santos C., Gomes I.L., Afonso G.S., Vieira R., Teles H.S.S. (2021). A Molecular Test Based on RT-LAMP for Rapid, Sensitive and Inexpensive Colorimetric Detection of SARS-CoV-2 in Clinical Samples. Sci. Rep..

[39] Broughton J.P., Deng X., Yu G., Fasching C.L., Servellita V., Singh J., Miao X., Streithorst J.A., Granados A., Sotomayor-Gonzalez A. (2020). CRISPR–Cas12-Based Detection of SARS-CoV-2. Nat. Biotechnol..

[40] Li Z., Yi Y., Luo X., Xiong N., Liu Y., Li S., Sun R., Wang Y., Hu B., Chen W. (2020). Development and Clinical Application of a Rapid IgM-IgG Combined Antibody Test for SARS-CoV-2 Infection Diagnosis. J. Med. Virol..

[41] Roda A., Cavalera S., Di Nardo F., Calabria D., Rosati S., Simoni P., Colitti B., Baggiani C., Roda M., Anfossi L. (2021). Dual Lateral Flow Optical/Chemiluminescence Immunosensors for the Rapid Detection of Salivary and Serum IgA in Patients with COVID-19 Disease. Biosens. Bioelectron..

[42] Theel E.S., Harring J., Hilgart H., Granger D., McAdam A.J. (2020). Performance Characteristics of Four High-Throughput Immunoassays for Detection of IgG Antibodies against SARS-CoV-2. J. Clin. Microbiol..

[43] Human SARS-CoV-2 Spike (Trimer) IgM ELISA Kit – Invitrogen. https://www.thermofisher.com/elisa/product/Human-SARS-CoV-2-Spike-Trimer-IgM-ELISA-Kit/BMS2324.

[44] Kudriashova I.B., Kirnos M.D., Vaniushin B.F. (1976). DNA-Methylase Activities from Animal Mitochondria and Nuclei: Different Specificity of DNA Methylation. Biokhimiia.

[45] Jing M., Bond R., Robertson L.J., Moore J., Kowalczyk A., Price R., Burns W., Nesbit M.A., McLaughlin J., Moore T. (2021). User Experience Analysis of AbC-19 Rapid Test via Lateral Flow Immunoassays for Self-Administrated SARS-CoV-2 Antibody Testing. Sci. Rep..

[46] COVID-19 Total Antibody ELISA Kit. https://www.biopanda.co.uk/php/products/elisa/covid19tabelisa.php.

[47] SARS-CoV-2 Antigen Rapid Test - Results in 15 Minutes | Kiweno. https://kiweno.com/en/sars-cov2-antigen-test/.

[48] Mertens P., De Vos N., Martiny D., Jassoy C., Mirazimi A., Cuypers L., den Wijngaert S., Monteil V., Melin P., Stoffels K. (2020). Development and Potential Usefulness of the COVID-19 Ag Respi-Strip Diagnostic Assay in a Pandemic Context. Front. Med..

[49] Use of SARS-CoV-2 Antigen-Detection Rapid Diagnostic Tests for COVID-19 Self-Testing. https://www.who.int/publications/i/item/WHO-2019-nCoV-Ag-RDTs-Self_testing-2022.1.

[50] Rashid Z.Z., Othman S.N., Samat M.N.A., Ali U.K., Wong K.K. (2020). Diagnostic Performance of COVID-19 Serology Assays. Malays. J. Pathol..

[51] Yüce M., Filiztekin E., Özkaya K.G. (2021). COVID-19 Diagnosis—A Review of Current Methods. Biosens. Bioelectron..

[52] Edward_holmes Novel 2019 Coronavirus Genome - SARS-CoV-2 Coronavirus – Virological. https://virological.org/t/novel-2019-coronavirus-genome/319.

[53] Hu B., Ge X., Wang L.-F., Shi Z. (2015). Bat Origin of Human Coronaviruses. Virol. J..

[54] Mostafa H.H., Fissel J.A., Fanelli B., Bergman Y., Gniazdowski V., Dadlani M., Carroll K.C., Colwell R.R., Simner P.J. (2020). Metagenomic Next-Generation Sequencing of Nasopharyngeal Specimens Collected from Confirmed and Suspect COVID-19 Patients. MBio.

[55] Zhang N., Wang L., Deng X., Liang R., Su M., He C., Hu L., Su Y., Ren J., Yu F. (2020). Recent Advances in the Detection of Respiratory Virus Infection in Humans. J. Med. Virol..

[56] Harper H., Burridge A., Winfield M., Finn A., Davidson A., Matthews D., Hutchings S., Vipond B., Jain N., The COVID-19 Genomics UK (COG-UK) Consortium (2021). Detecting SARS-CoV-2 Variants with SNP Genotyping. PLoS ONE.

[57] Pan Y., Guan H., Zhou S., Wang Y., Li Q., Zhu T., Hu Q., Xia L. (2020). Initial CT Findings and Temporal Changes in Patients with the Novel Coronavirus Pneumonia (2019-NCoV): A Study of 63 Patients in Wuhan, China. Eur. Radiol..

[58] Chung M., Bernheim A., Mei X., Zhang N., Huang M., Zeng X., Cui J., Xu W., Yang Y., Fayad Z.A. (2020). CT Imaging Features of 2019 Novel Coronavirus (2019-NCoV). Radiology.

[59] Ooi G.C., Khong P.L., Müller N.L., Yiu W.C., Zhou L.J., Ho J.C.M., Lam B., Nicolaou S., Tsang K.W.T. (2004). Severe Acute Respiratory Syndrome: Temporal Lung Changes at Thin-Section CT in 30 Patients. Radiology.

[60] Wijmans L., Mooij-Kalverda K.A., Bos L., Goorsenberg A., Smit M., Van Den Berk I., De Bruin D., Bonta P., Schultz M., Annema J. (2019). Optical Coherence Tomography (OCT) in Patients with Acute Respiratory Failure on the ICU. Eur. Respir. J..

[61] Steinberg I., Huland D.M., Vermesh O., Frostig H.E., Tummers W.S., Gambhir S.S. (2019). Photoacoustic Clinical Imaging. Photoacoustics.

[62] Shi R., Ma W., Wu Q., Zhang B., Song Y., Guo Q., Xiao W., Wang Y., Zheng W. (2003). Design and Application of 60mer Oligonucleotide Microarray in SARS Coronavirus Detection. Chin. Sci. Bull..

[63] de Kleber S.L.L., Volker H., Nicolas R., Marcus P., Felix D.J., Sabue M., Leo P., Sigrid B., Jan H.B., Laurent K. (2007). Generic Detection of Coronaviruses and Differentiation at the Prototype Strain Level by Reverse Transcription-PCR and Nonfluorescent Low-Density Microarray. J. Clin. Microbiol..

[64] Hardick J., Metzgar D., Risen L., Myers C., Balansay M., Malcom T., Rothman R., Gaydos C. (2018). Initial Performance Evaluation of a Spotted Array Mobile Analysis Platform (MAP) for the Detection of Influenza A/B, RSV, and MERS Coronavirus. Diagn. Microbiol. Infect. Dis..

[65] Corman V.M., Landt O., Kaiser M., Molenkamp R., Meijer A., Chu D.K., Bleicker T., Brünink S., Schneider J., Schmidt M.L. (2020). Detection of 2019 Novel Coronavirus (2019-NCoV) by Real-Time RT-PCR. Euro Surveill..

[66] Adachi D., Johnson G., Draker R., Ayers M., Mazzulli T., Talbot P.J., Tellier R. (2004). Comprehensive Detection and Identification of Human Coronaviruses, Including the SARS-Associated Coronavirus, with a Single RT-PCR Assay. J. Virol. Methods.

[67] Setianingsih T.Y., Wiyatno A., Hartono T.S., Hindawati E., Dewantari A.K., Myint K.S., Lisdawati V., Safari D. (2019). Detection of Multiple Viral Sequences in the Respiratory Tract Samples of Suspected Middle East Respiratory Syndrome Coronavirus Patients in Jakarta, Indonesia 2015–2016. Int. J. Infect. Dis..

[68] Corman V.M., Eckerle I., Bleicker T., Zaki A., Landt O., Eschbach-Bludau M., van Boheemen S., Gopal R., Ballhause M., Bestebroer T.M. (2012). Detection of a Novel Human Coronavirus by Real-Time Reverse-Transcription Polymerase Chain Reaction. Eurosurveillance.

[69] Lu X., Whitaker B., Sakthivel S.K.K., Kamili S., Rose L.E., Lowe L., Mohareb E., Elassal E.M., Al-sanouri T., Haddadin A. (2014). Real-Time Reverse Transcription-PCR Assay Panel for Middle East Respiratory Syndrome Coronavirus. J. Clin. Microbiol..

[70] Bartholomew R.A., Hutchison J.R., Straub T.M., Call D.R. (2016). PCR, Real-Time PCR, Digital PCR, and Isothermal Amplification. Manual of Environmental Microbiology.

[71] Lu X., Wang L., Sakthivel S.K., Whitaker B., Murray J., Kamili S., Lynch B., Malapati L., Burke S.A., Harcourt J. US CDC Real-Time Reverse Transcription PCR Panel for Detection of Severe Acute Respiratory Syndrome Coronavirus 2 - Volume 26, Number 8—August 2020 - Emerging Infectious Diseases Journal – CDC. https://wwwnc.cdc.gov/eid/article/26/8/20-1246_article.

[72] Pyrc K., Milewska A., Potempa J. (2011). Development of Loop-Mediated Isothermal Amplification Assay for Detection of Human Coronavirus-NL63. J. Virol. Methods.

[73] Poon L.L.M., Leung C.S.W., Tashiro M., Chan K.H., Wong B.W.Y., Yuen K.Y., Guan Y., Peiris J.S.M. (2004). Rapid Detection of the Severe Acute Respiratory Syndrome (SARS) Coronavirus by a Loop-Mediated Isothermal Amplification Assay. Clin. Chem..

[74] Zou M., Su F., Zhang R., Jiang X., Xiao H., Yan X., Yang C., Fan X., Wu G. (2021). Rapid Point-of-Care Testing for SARS-CoV-2 Virus Nucleic Acid Detection by an Isothermal and Nonenzymatic Signal Amplification System Coupled with a Lateral Flow Immunoassay Strip. Sens. Actuators B Chem..

[75] Shi H., Han X., Zheng C. (2020). Evolution of CT Manifestations in a Patient Recovered from 2019 Novel Coronavirus (2019-NCoV) Pneumonia in Wuhan, China. Radiology.

[76] Park T.J., Lee S.J., Kim D.-K., Heo N.S., Park J.Y., Lee S.Y. (2012). Development of Label-Free Optical Diagnosis for Sensitive Detection of Influenza Virus with Genetically Engineered Fusion Protein. Talanta.

[77] Njiru Z.K. (2012). Loop-Mediated Isothermal Amplification Technology: Towards Point of Care Diagnostics. PLoS Negl. Trop. Dis..

[78] Yang W., Dang X., Wang Q., Xu M., Zhao Q., Zhou Y., Zhao H., Wang L., Xu Y., Wang J. (2020). Rapid Detection of SARS-CoV-2 Using Reverse Transcription RT-LAMP Method. medRxiv.

[79] Mohammadniaei M., Yoon J., Choi H.K., Placide V., Bharate B.G., Lee T., Choi J.-W. (2019). Multifunctional Nanobiohybrid Material Composed of Ag@Bi2Se3/RNA Three-Way Junction/MiRNA/Retinoic Acid for Neuroblastoma Differentiation. ACS Appl. Mater. Interfaces.

[80] Park G.-S., Ku K., Baek S.-H., Kim S.-J., Il Kim S., Kim B.-T., Maeng J.-S. (2020). Development of Reverse Transcription Loop-Mediated Isothermal Amplification Assays Targeting Severe Acute Respiratory Syndrome Coronavirus 2 (SARS-CoV-2). J. Mol. Diagn..

[81] Shirato K., Semba S., El-Kafrawy S.A., Hassan A.M., Tolah A.M., Takayama I., Kageyama T., Notomi T., Kamitani W., Matsuyama S. (2018). Development of Fluorescent Reverse Transcription Loop-Mediated Isothermal Amplification (RT-LAMP) Using Quenching Probes for the Detection of the Middle East Respiratory Syndrome Coronavirus. J. Virol. Methods.

[82] Xu G., Lai M., Wilson R., Glidle A., Reboud J., Cooper J.M. (2019). Branched Hybridization Chain Reaction—Using Highly Dimensional DNA Nanostructures for Label-Free, Reagent-Less, Multiplexed Molecular Diagnostics. Microsystems Nanoeng..

[83] Wang B., Thachuk C., Ellington A.D., Winfree E., Soloveichik D. (2018). Effective Design Principles for Leakless Strand Displacement Systems. Proc. Natl. Acad. Sci. USA.

[84] Yin P., Choi H.M.T., Calvert C.R., Pierce N.A. (2008). Programming Biomolecular Self-Assembly Pathways. Nature.

[85] Li B., Chen X., Ellington A.D. (2012). Adapting Enzyme-Free DNA Circuits to the Detection of Loop-Mediated Isothermal Amplification Reactions. Anal. Chem..

[86] Jiao J., Duan C., Xue L., Liu Y., Sun W., Xiang Y. (2020). DNA Nanoscaffold-Based SARS-CoV-2 Detection for COVID-19 Diagnosis. Biosens. Bioelectron..

[87] Bhadra S., Riedel T.E., Lakhotia S., Tran N.D., Ellington A.D. (2020). High-Surety Isothermal Amplification and Detection of SARS-CoV-2, Including with Crude Enzymes. bioRxiv.

[88] Do J.Y., Jeong J.Y., Hong C.A. (2021). Catalytic Hairpin DNA Assembly-Based Chemiluminescent Assay for the Detection of Short SARS-CoV-2 Target CDNA. Talanta.

[89] Christensen U.B., Wamberg M., El-Essawy F.A.G., Ismail A.E., Nielsen C.B., Filichev V.V., Jessen C.H., Petersen M., Pedersen E.B. (2004). Intercalating Nucleic Acids: The Influence of Linker Length and Intercalator Type on Their Duplex Stabilities. Nucleosides. Nucleotides Nucleic Acids.

[90] Shirato K., Yano T., Senba S., Akachi S., Kobayashi T., Nishinaka T., Notomi T., Matsuyama S. (2014). Detection of Middle East Respiratory Syndrome Coronavirus Using Reverse Transcription Loop-Mediated Isothermal Amplification (RT-LAMP). Virol. J..

[91] Mautner L., Baillie C.-K., Herold H.M., Volkwein W., Guertler P., Eberle U., Ackermann N., Sing A., Pavlovic M., Goerlich O. (2020). Rapid Point-of-Care Detection of SARS-CoV-2 Using Reverse Transcription Loop-Mediated Isothermal Amplification (RT-LAMP). Virol. J..

[92] Rohaim M.A., Clayton E., Sahin I., Vilela J., Khalifa M.E., Al-Natour M.Q., Bayoumi M., Poirier A.C., Branavan M., Tharmakulasingam M. (2020). Artificial Intelligence-Assisted Loop Mediated Isothermal Amplification (AI-LAMP) for Rapid Detection of SARS-CoV-2. Viruses.

[93] Mohammadniaei M., Zhang M., Ashley J., Christensen U.B., Friis-Hansen L.J., Gregersen R., Lisby J.G., Benfield T.L., Nielsen F.E., Henning Rasmussen J. (2021). A Non-Enzymatic, Isothermal Strand Displacement and Amplification Assay for Rapid Detection of SARS-CoV-2 RNA. Nat. Commun..

[94] Wu S., Liu X., Ye S., Liu J., Zheng W., Dong X., Yin X. (2021). Colorimetric Isothermal Nucleic Acid Detection of SARS-CoV-2 with Dye Combination. Heliyon.

[95] Liang P., Xu Y., Zhang X., Ding C., Huang R., Zhang Z., Lv J., Xie X., Chen Y., Li Y. (2015). CRISPR/Cas9-Mediated Gene Editing in Human Tripronuclear Zygotes. Protein Cell.

[96] Lin X., Liu Y., Chemparathy A., Pande T., La Russa M., Qi L.S. (2021). A Comprehensive Analysis and Resource to Use CRISPR-Cas13 for Broad-Spectrum Targeting of RNA Viruses. Cell Reports Med..

[97] Broughton J.P., Deng X., Yu G., Fasching C.L., Singh J., Streithorst J., Granados A., Sotomayor-Gonzalez A., Zorn K., Gopez A. (2020). Rapid Detection of 2019 Novel Coronavirus SARS-CoV-2 Using a CRISPR-Based DETECTR Lateral Flow Assay. medRxiv Prepr. Serv. Health Sci..

[98] Ding X., Yin K., Li Z., Liu C. (2020). All-in-One Dual CRISPR-Cas12a (AIOD-CRISPR) Assay: A Case for Rapid, Ultrasensitive and Visual Detection of Novel Coronavirus SARS-CoV-2 and HIV Virus. bioRxiv.

[99] Kushawah G., Hernandez-Huertas L., Abugattas-Nuñez del Prado J., Martinez-Morales J.R., DeVore M.L., Hassan H., Moreno-Sanchez I., Tomas-Gallardo L., Diaz-Moscoso A., Monges D.E. (2020). CRISPR-Cas13d Induces Efficient MRNA Knockdown in Animal Embryos. Dev. Cell.

[100] Rauch J.N., Valois E., Solley S.C., Braig F., Lach R.S., Audouard M., Ponce-Rojas J.C., Costello M.S., Baxter N.J., Kosik K.S. (2020). A Scalable, Easy-to-Deploy, Protocol for Cas13-Based Detection of SARS-CoV-2 Genetic Material. bioRxiv.

[101] Mustafa M.I., Makhawi A.M., Kraft C.S. (2021). SHERLOCK and DETECTR: CRISPR-Cas Systems as Potential Rapid Diagnostic Tools for Emerging Infectious Diseases. J. Clin. Microbiol..

[102] Santiago-Frangos A., Hall L.N., Nemudraia A., Nemudryi A., Krishna P., Wiegand T., Wilkinson R.A., Snyder D.T., Hedges J.F., Cicha C. (2021). Intrinsic Signal Amplification by Type III CRISPR-Cas Systems Provides a Sequence-Specific SARS-CoV-2 Diagnostic. Cell Rep. Med..

[103] Fozouni P., Son S., Díaz de León Derby M., Knott G.J., Gray C.N., D’Ambrosio M.V., Zhao C., Switz N.A., Kumar G.R., Stephens S.I. (2021). Amplification-Free Detection of SARS-CoV-2 with CRISPR-Cas13a and Mobile Phone Microscopy. Cell.

[104] Guo X., Guo Z., Duan C., Chen Z., Wang G., Lu Y., Li M., Lu J. (2020). Long-Term Persistence of IgG Antibodies in SARS-CoV Infected Healthcare Workers. medRxiv.

[105] Wang W., Xu Y., Gao R., Lu R., Han K., Wu G., Tan W. (2020). Detection of SARS-CoV-2 in Different Types of Clinical Specimens. JAMA.

[106] Green D.A., Zucker J., Westblade L.F., Whittier S., Rennert H., Velu P., Craney A., Cushing M., Liu D., Sobieszczyk M.E. (2020). Clinical Performance of SARS-CoV-2 Molecular Tests. J. Clin. Microbiol..

[107] Tahamtan A., Ardebili A. (2020). Real-Time RT-PCR in COVID-19 Detection: Issues Affecting the Results. Expert Rev. Mol. Diagn..

[108] Loeffelholz M.J., Pong D.L., Pyles R.B., Xiong Y., Miller A.L., Bufton K.K., Chonmaitree T. (2011). Comparison of the FilmArray Respiratory Panel and Prodesse Real-Time PCR Assays for Detection of Respiratory Pathogens. J. Clin. Microbiol..

[109] (2021). CRISPR-Based Portable COVID Tests. Nat. Biotechnol..

[110] Chandrasekaran A.R. (2017). DNA Nanobiosensors: An Outlook on Signal Readout Strategies. J. Nanomater..

[111] Rajendran V.K., Bakthavathsalam P., Bergquist P.L., Sunna A. (2019). A Portable Nucleic Acid Detection System Using Natural Convection Combined with a Smartphone. Biosens. Bioelectron..

[112] Paradiso A.V., De Summa S., Loconsole D., Procacci V., Sallustio A., Centrone F., Silvestris N., Cafagna V., De Palma G., Tufaro A. (2020). Rapid Serological Assays and SARS-CoV-2 Real-Time Polymerase Chain Reaction Assays for the Detection of SARS-CoV-2: Comparative Study. J. Med. Internet. Res..

[113] Huang C., Tan Z., Zhao K., Zou W., Wang H., Gao H., Sun S., Bu D., Chai W., Li Y. (2021). The Effect of N-Glycosylation of SARS-CoV-2 Spike Protein on the Virus Interaction with the Host Cell ACE2 Receptor. iScience.

[114] Robson B. (2020). COVID-19 Coronavirus Spike Protein Analysis for Synthetic Vaccines, a Peptidomimetic Antagonist, and Therapeutic Drugs, and Analysis of a Proposed Achilles’ Heel Conserved Region to Minimize Probability of Escape Mutations and Drug Resistance. Comput. Biol. Med..

[115] Jalkanen P., Pasternack A., Maljanen S., Melén K., Kolehmainen P., Huttunen M., Lundberg R., Tripathi L., Khan H., Ritvos M.A. (2021). A Combination of N and S Antigens With IgA and IgG Measurement Strengthens the Accuracy of SARS-CoV-2 Serodiagnostics. J. Infect. Dis..

[116] Singh P., Chakraborty R., Marwal R., Radhakrishan V.S., Bhaskar A.K., Vashisht H., Dhar M.S., Pradhan S., Ranjan G., Imran M. (2020). A Rapid and Sensitive Method to Detect SARS-CoV-2 Virus Using Targeted-Mass Spectrometry. J. Proteins Proteomics.

[117] Dogan M., Kozhaya L., Placek L., Gunter C., Yigit M., Hardy R., Plassmeyer M., Coatney P., Lillard K., Bukhari Z. (2021). SARS-CoV-2 Specific Antibody and Neutralization Assays Reveal the Wide Range of the Humoral Immune Response to Virus. Commun. Biol..

[118] Frank S.A. (2002). Specificity and Cross-Reactivity. Immunology and Evolution of Infectious Disease.

[119] Zhou Y., Chen Y., Liu W., Fang H., Li X., Hou L., Liu Y., Lai W., Huang X., Xiong Y. (2021). Development of a Rapid and Sensitive Quantum Dot Nanobead-Based Double-Antigen Sandwich Lateral Flow Immunoassay and Its Clinical Performance for the Detection of SARS-CoV-2 Total Antibodies. Sens. Actuators B Chem..

[120] Jung C., Levy C., Varon E., Biscardi S., Batard C., Wollner A., Deberdt P., Sellam A., Hau I., Cohen R. (2021). Diagnostic Accuracy of SARS-CoV-2 Antigen Detection Test in Children: A Real-Life Study. Front. Pediatr..

[121] Lau S.K.P., Che X.-Y., Yuen K.-Y., Wong B.H.L., Cheng V.C.C., Woo G.K.S., Hung I.F.N., Poon R.W.S., Chan K.-H., Peiris J.S.M. (2005). SARS Coronavirus Detection Methods. Emerg. Infect. Dis. J..

[122] Lv L., Xie X., Gong Q., Feng R., Guo X., Su B., Chen L. (2020). Transcriptional Difference between SARS-COV-2 and Other Human Coronaviruses Revealed by Sub-Genomic RNA Profiling. bioRxiv.

[123] Liu Q., Cheng S., Chen R., Ke J., Liu Y., Li Y., Feng W., Li F., Liu C., Geva E. (2020). An Isothermal Amplification Reactor with an Integrated Isolation Membrane for Point-of-Care Detection of Infectious Diseases. Analyst.

[124] Liu Q., Cheng S., Chen R., Ke J., Liu Y., Li Y., Feng W., Li F. (2020). Near-Infrared Lanthanide-Doped Nanoparticles for a Low Interference Lateral Flow Immunoassay Test. ACS Appl. Mater. Interfaces.

[125] Charles A., Janeway J., Travers P., Walport M., Shlomchik M.J. (2001). The Distribution and Functions of Immunoglobulin Isotypes. Immunobiology: The Immune System in Health and Disease.

[126] Long Q.-X., Liu B.-Z., Deng H.-J., Wu G.-C., Deng K., Chen Y.-K., Liao P., Qiu J.-F., Lin Y., Cai X.-F. (2020). Antibody Responses to SARS-CoV-2 in Patients with COVID-19. Nat. Med..

[127] To K.K.-W., Tsang O.T.-Y., Leung W.-S., Tam A.R., Wu T.-C., Lung D.C., Yip C.C.-Y., Cai J.-P., Chan J.M.-C., Chik T.S.-H. (2020). Temporal Profiles of Viral Load in Posterior Oropharyngeal Saliva Samples and Serum Antibody Responses during Infection by SARS-CoV-2: An Observational Cohort Study. Lancet Infect. Dis..

[128] Bhalla N., Jolly P., Formisano N., Estrela P. (2016). Introduction to Biosensors. Essays Biochem..

[129] Land K.J., Boeras D.I., Chen X.-S., Ramsay A.R., Peeling R.W. (2019). REASSURED Diagnostics to Inform Disease Control Strategies, Strengthen Health Systems and Improve Patient Outcomes. Nat. Microbiol..

[130] Tripathy S., Singh S.G. (2020). Label-Free Electrochemical Detection of DNA Hybridization: A Method for COVID-19 Diagnosis. Trans. Indian Natl. Acad. Eng..

[131] Qiu G., Gai Z., Tao Y., Schmitt J., Kullak-Ublick G.A., Wang J. (2020). Dual-Functional Plasmonic Photothermal Biosensors for Highly Accurate Severe Acute Respiratory Syndrome Coronavirus 2 Detection. ACS Nano.

[132] Moitra P., Alafeef M., Dighe K., Frieman M.B., Pan D. (2020). Selective Naked-Eye Detection of SARS-CoV-2 Mediated by N Gene Targeted Antisense Oligonucleotide Capped Plasmonic Nanoparticles. ACS Nano.

[133] Mavrikou S., Moschopoulou G., Tsekouras V., Kintzios S. (2020). Development of a Portable, Ultra-Rapid and Ultra-Sensitive Cell-Based Biosensor for the Direct Detection of the SARS-CoV-2 S1 Spike Protein Antigen. Sensors.

[134] Huang L., Ding L., Zhou J., Chen S., Chen F., Zhao C., Xu J., Hu W., Ji J., Xu H. (2021). One-Step Rapid Quantification of SARS-CoV-2 Virus Particles via Low-Cost Nanoplasmonic Sensors in Generic Microplate Reader and Point-of-Care Device. Biosens. Bioelectron..

[135] Seo G., Lee G., Kim M.J., Baek S.-H., Choi M., Ku K.B., Lee C.-S., Jun S., Park D., Kim H.G. (2020). Rapid Detection of COVID-19 Causative Virus (SARS-CoV-2) in Human Nasopharyngeal Swab Specimens Using Field-Effect Transistor-Based Biosensor. ACS Nano.

[136] Zhang R., Ke X., Gu Y., Liu H., Sun X. (2020). Whole Genome Identification of Potential G-Quadruplexes and Analysis of the G-Quadruplex Binding Domain for SARS-CoV-2. Front. Genet..

[137] Parisi O.I., Dattilo M., Patitucci F., Malivindi R., Pezzi V., Perrotta I., Ruffo M., Amone F., Puoci F. (2020). ‘Monoclonal-Type’ Plastic Antibodies for SARS-CoV-2 Based on Molecularly Imprinted Polymers. bioRxiv.

[138] Mahari S., Roberts A., Shahdeo D., Gandhi S. (2020). ECovSens-Ultrasensitive Novel In-House Built Printed Circuit Board Based Electrochemical Device for Rapid Detection of NCovid-19 Antigen, a Spike Protein Domain 1 of SARS-CoV-2. bioRxiv.

[139] Vadlamani B.S., Uppal T., Verma S.C., Misra M. (2020). Functionalized TiO2 Nanotube-Based Electrochemical Biosensor for Rapid Detection of SARS-CoV-2. Sensors.

[140] Lee J.-H., Choi M., Jung Y., Lee S.K., Lee C.-S., Kim J., Kim J., Kim N.H., Kim B.-T., Kim H.G. (2021). A Novel Rapid Detection for SARS-CoV-2 Spike 1 Antigens Using Human Angiotensin Converting Enzyme 2 (ACE2). Biosens. Bioelectron..

[141] Chen Z., Zhang Z., Zhai X., Li Y., Lin L., Zhao H., Bian L., Li P., Yu L., Wu Y. (2020). Rapid and Sensitive Detection of Anti-SARS-CoV-2 IgG, Using Lanthanide-Doped Nanoparticles-Based Lateral Flow Immunoassay. Anal. Chem..

[142] Zeng L., Li Y., Liu J., Guo L., Wang Z., Xu X., Song S., Hao C., Liu L., Xin M. (2020). Rapid, Ultrasensitive and Highly Specific Biosensor for the Diagnosis of SARS-CoV-2 in Clinical Blood Samples. Mater. Chem. Front..

[143] Cady N.C., Tokranova N., Minor A., Nikvand N., Strle K., Lee W.T., Page W., Guignon E., Pilar A., Gibson G.N. (2021). Multiplexed Detection and Quantification of Human Antibody Response to COVID-19 Infection Using a Plasmon Enhanced Biosensor Platform. Biosens. Bioelectron..

[144] Funari R., Chu K.-Y., Shen A.Q. (2020). Detection of Antibodies against SARS-CoV-2 Spike Protein by Gold Nanospikes in an Opto-Microfluidic Chip. Biosens. Bioelectron..

[145] Rodriguez-Manzano J., Malpartida-Cardenas K., Moser N., Pennisi I., Cavuto M., Miglietta L., Moniri A., Penn R., Satta G., Randell P. (2021). Handheld Point-of-Care System for Rapid Detection of SARS-CoV-2 Extracted RNA in under 20 Min. ACS Cent. Sci..

[146] Kim H., Abbas N., Shin S. (2021). A Rapid Diagnosis of SARS-CoV-2 Using DNA Hydrogel Formation on Microfluidic Pores. Biosens. Bioelectron..

[147] Ramachandran A., Huyke D.A., Sharma E., Sahoo M.K., Huang C., Banaei N., Pinsky B.A., Santiago J.G. (2020). Electric Field-Driven Microfluidics for Rapid CRISPR-Based Diagnostics and Its Application to Detection of SARS-CoV-2. Proc. Natl. Acad. Sci. USA.

[148] Wang D., He S., Wang X., Yan Y., Liu J., Wu S., Liu S., Lei Y., Chen M., Li L. (2020). Rapid Lateral Flow Immunoassay for the Fluorescence Detection of SARS-CoV-2 RNA. Nat. Biomed. Eng..

[149] Huang X., Aguilar Z.P., Xu H., Lai W., Xiong Y. (2016). Membrane-Based Lateral Flow Immunochromatographic Strip with Nanoparticles as Reporters for Detection: A Review. Biosens. Bioelectron..

[150] Wang X., Wu X., Lu Z., Tao X. (2020). Comparative Study of Time-Resolved Fluorescent Nanobeads, Quantum Dot Nanobeads and Quantum Dots as Labels in Fluorescence Immunochromatography for Detection of Aflatoxin B1 in Grains. Biomolecules.

[151] Vaitukaitis J.L., Braunstein G.D., Ross G.T. (1972). A Radioimmunoassay Which Specifically Measures Human Chorionic Gonadotropin in the Presence of Human Luteinizing Hormone. Am. J. Obstet. Gynecol..

[152] Zhu X., Wang X., Han L., Chen T., Wang L., Li H., Li S., He L., Fu X., Chen S. (2020). Multiplex Reverse Transcription Loop-Mediated Isothermal Amplification Combined with Nanoparticle-Based Lateral Flow Biosensor for the Diagnosis of COVID-19. Biosens. Bioelectron..

[153] Yan C., Cui J., Huang L., Du B., Chen L., Xue G., Li S., Zhang W., Zhao L., Sun Y. (2020). Rapid and Visual Detection of 2019 Novel Coronavirus (SARS-CoV-2) by a Reverse Transcription Loop-Mediated Isothermal Amplification Assay. Clin. Microbiol. Infect..

[154] Xiong E., Jiang L., Tian T., Hu M., Yue H., Huang M., Lin W., Jiang Y., Zhu D., Zhou X. (2021). Simultaneous Dual-Gene Diagnosis of SARS-CoV-2 Based on CRISPR/Cas9-Mediated Lateral Flow Assay. Angew. Chem. Int. Ed..

[155] Zhang C., Zheng T., Wang H., Chen W., Huang X., Liang J., Qiu L., Han D., Tan W. (2021). Rapid One-Pot Detection of SARS-CoV-2 Based on a Lateral Flow Assay in Clinical Samples. Anal. Chem..

[156] Qian J., Boswell S.A., Chidley C., Lu Z., Pettit M.E., Gaudio B.L., Fajnzylber J.M., Ingram R.T., Ward R.H., Li J.Z. (2020). An Enhanced Isothermal Amplification Assay for Viral Detection. Nat. Commun..

[157] Peng T., Sui Z., Huang Z., Xie J., Wen K., Zhang Y., Huang W., Mi W., Peng K., Dai X. (2021). Point-of-Care Test System for Detection of Immunoglobulin-G and -M against Nucleocapsid Protein and Spike Glycoprotein of SARS-CoV-2. Sens. Actuators B Chem..

[158] Bayin Q., Huang L., Ren C., Fu Y., Ma X., Guo J. (2021). Anti-SARS-CoV-2 IgG and IgM Detection with a GMR Based LFIA System. Talanta.

[159] Grant B.D., Anderson C.E., Williford J.R., Alonzo L.F., Glukhova V.A., Boyle D.S., Weigl B.H., Nichols K.P. (2020). SARS-CoV-2 Coronavirus Nucleocapsid Antigen-Detecting Half-Strip Lateral Flow Assay Toward the Development of Point of Care Tests Using Commercially Available Reagents. Anal. Chem..

[160] Acquah C., Jeevanandam J., Tan K.X., Danquah M.K. (2021). Engineered Aptamers for Enhanced COVID-19 Theranostics. Cell. Mol. Bioeng..

[161] Zhang L., Fang X., Liu X., Ou H., Zhang H., Wang J., Li Q., Cheng H., Zhang W., Luo Z. (2020). Discovery of Sandwich Type COVID-19 Nucleocapsid Protein DNA Aptamers. Chem. Commun..

[162] Devi A., Chaitanya N.S.N. (2022). Designing of Peptide Aptamer Targeting the Receptor-Binding Domain of Spike Protein of SARS-CoV-2: An in Silico Study. Mol. Divers..

[163] Li J., Lillehoj P.B. (2021). Microfluidic Magneto Immunosensor for Rapid, High Sensitivity Measurements of SARS-CoV-2 Nucleocapsid Protein in Serum. ACS Sensors.

[164] Nehra A., Pal Singh K. (2015). Current Trends in Nanomaterial Embedded Field Effect Transistor-Based Biosensor. Biosens. Bioelectron..

[165] Janissen R., Sahoo P.K., Santos C.A., da Silva A.M., von Zuben A.A.G., Souto D.E.P., Costa A.D.T., Celedon P., Zanchin N.I.T., Almeida D.B. (2017). InP Nanowire Biosensor with Tailored Biofunctionalization: Ultrasensitive and Highly Selective Disease Biomarker Detection. Nano Lett..

[166] Chen Y., Ren R., Pu H., Guo X., Chang J., Zhou G., Mao S., Kron M., Chen J. (2017). Field-Effect Transistor Biosensor for Rapid Detection of Ebola Antigen. Sci. Rep..

[167] Geim A.K., Novoselov K.S. (2009). The Rise of Graphene. Nanoscience and Technology.

[168] Lin Q., Wen D., Wu J., Liu L., Wu W., Fang X., Kong J. (2020). Microfluidic Immunoassays for Sensitive and Simultaneous Detection of IgG/IgM/Antigen of SARS-CoV-2 within 15 Min. Anal. Chem..

[169] Laksanasopin T., Guo T.W., Nayak S., Sridhara A.A., Xie S., Olowookere O.O., Cadinu P., Meng F., Chee N.H., Kim J. (2015). A Smartphone Dongle for Diagnosis of Infectious Diseases at the Point of Care. Sci. Transl. Med..

[170] Garcia-Cordero J.L., Maerkl S.J. (2015). Mechanically Induced Trapping of Molecular Interactions and Its Applications. J. Lab. Autom..

[171] Swank Z., Michielin G., Yip H.M., Cohen P., Andrey D.O., Vuilleumier N., Kaiser L., Eckerle I., Meyer B., Maerkl S.J. (2021). A High-Throughput Microfluidic Nanoimmunoassay for Detecting Anti–SARS-CoV-2 Antibodies in Serum or Ultralow-Volume Blood Samples. Proc. Natl. Acad. Sci. USA.

[172] Mettakoonpitak J., Boehle K., Nantaphol S., Teengam P., Adkins J.A., Srisa-Art M., Henry C.S. (2016). Electrochemistry on Paper-Based Analytical Devices: A Review. Electroanalysis.

[173] Yetisen A.K., Akram M.S., Lowe C.R. (2013). Paper-Based Microfluidic Point-of-Care Diagnostic Devices. Lab Chip.

[174] Teengam P., Siangproh W., Tuantranont A., Vilaivan T., Chailapakul O., Henry C.S. (2017). Multiplex Paper-Based Colorimetric DNA Sensor Using Pyrrolidinyl Peptide Nucleic Acid-Induced AgNPs Aggregation for Detecting MERS-CoV, MTB, and HPV Oligonucleotides. Anal. Chem..

[175] Han G.-R., Ki H., Kim M.-G. (2020). Automated, Universal, and Mass-Producible Paper-Based Lateral Flow Biosensing Platform for High-Performance Point-of-Care Testing. ACS Appl. Mater. Interfaces.

[176] Nguyen V.-T., Song S., Park S., Joo C. (2020). Recent Advances in High-Sensitivity Detection Methods for Paper-Based Lateral-Flow Assay. Biosens. Bioelectron..

[177] Pilavaki E., Demosthenous A. (2017). Optimized Lateral Flow Immunoassay Reader for the Detection of Infectious Diseases in Developing Countries. Sensors.

[178] Tabish T.A., Hamblin M.R. (2020). Multivalent Nanomedicines to Treat COVID-19: A Slow Train Coming. Nano Today.

[179] Farooq T., Adeel M., He Z., Umar M., Shakoor N., da Silva W., Elmer W., White J.C., Rui Y. (2021). Nanotechnology and Plant Viruses: An Emerging Disease Management Approach for Resistant Pathogens. ACS Nano.

[180] Amin M.T., Alazba A.A., Manzoor U. (2014). A Review of Removal of Pollutants from Water/Wastewater Using Different Types of Nanomaterials. Adv. Mater. Sci. Eng..

[181] Xiang J., Yan M., Li H., Liu T., Lin C., Huang S., Shen C. (2020). Evaluation of Enzyme-Linked Immunoassay and Colloidal Gold-Immunochromatographic Assay Kit for Detection of Novel Coronavirus (SARS-Cov-2) Causing an Outbreak of Pneumonia (COVID-19). medRxiv.

[182] Zodrow K., Brunet L., Mahendra S., Li D., Zhang A., Li Q., Alvarez P.J.J. (2009). Polysulfone Ultrafiltration Membranes Impregnated with Silver Nanoparticles Show Improved Biofouling Resistance and Virus Removal. Water Res..

[183] Chen H., Huang M., Liu Y., Meng L., Ma M. (2020). Functionalized Electrospun Nanofiber Membranes for Water Treatment: A Review. Sci. Total Environ..

[184] Chen X., Zhou Q., Li S., Yan H., Chang B., Wang Y., Dong S. (2021). Rapid and Visual Detection of SARS-CoV-2 Using Multiplex Reverse Transcription Loop-Mediated Isothermal Amplification Linked With Gold Nanoparticle-Based Lateral Flow Biosensor. Front. Cell. Infect. Microbiol..

[185] Karakuş E., Erdemir E., Demirbilek N., Liv L. (2021). Colorimetric and Electrochemical Detection of SARS-CoV-2 Spike Antigen with a Gold Nanoparticle-Based Biosensor. Anal. Chim. Acta.

[186] Han H., Wang C., Yang X., Zheng S., Cheng X., Liu Z., Zhao B., Xiao R. (2022). Rapid Field Determination of SARS-CoV-2 by a Colorimetric and Fluorescent Dual-Functional Lateral Flow Immunoassay Biosensor. Sens. Actuators B Chem..

[187] Wandtke T., Woźniak J., Kopiński P. (2015). Aptamers in Diagnostics and Treatment of Viral Infections. Viruses.

[188] Ahn D.-G., Jeon I.-J., Kim J.D., Song M.-S., Han S.-R., Lee S.-W., Jung H., Oh J.-W. (2009). RNA Aptamer-Based Sensitive Detection of SARS Coronavirus Nucleocapsid Protein. Analyst.

[189] Shu Y., Shu D., Haque F., Guo P. (2013). Fabrication of PRNA Nanoparticles to Deliver Therapeutic RNAs and Bioactive Compounds into Tumor Cells. Nat. Protoc..

[190] Dey L., Chakraborty S., Mukhopadhyay A. (2020). Machine Learning Techniques for Sequence-Based Prediction of Viral–Host Interactions between SARS-CoV-2 and Human Proteins. Biomed. J..

[191] Zhang Y., Jiang H., Ye T., Juhas M. (2021). Deep Learning for Imaging and Detection of Microorganisms. Trends Microbiol..

[192] Layqah L.A., Eissa S. (2019). An Electrochemical Immunosensor for the Corona Virus Associated with the Middle East Respiratory Syndrome Using an Array of Gold Nanoparticle-Modified Carbon Electrodes. Microchim. Acta.

[193] Young B.E., Ong S.W.X., Kalimuddin S., Low J.G., Tan S.Y., Loh J., Ng O.-T., Marimuthu K., Ang L.W., Mak T.M. (2020). Epidemiologic Features and Clinical Course of Patients Infected With SARS-CoV-2 in Singapore. JAMA.

[194] Yoo H., Shin J., Sim J., Cho H., Hong S. (2020). Reusable Surface Plasmon Resonance Biosensor Chip for the Detection of H1N1 Influenza Virus. Biosens. Bioelectron..

[195] Estmer Nilsson C., Abbas S., Bennemo M., Larsson A., Hämäläinen M.D., Frostell-Karlsson Å. (2010). A Novel Assay for Influenza Virus Quantification Using Surface Plasmon Resonance. Vaccine.

[196] Garcia B.H., Goodman R.M. (2008). Use of Surface Plasmon Resonance Imaging to Study Viral RNA:Protein Interactions. J. Virol. Methods.

[197] Park T.J., Hyun M.S., Lee H.J., Lee S.Y., Ko S. (2009). A Self-Assembled Fusion Protein-Based Surface Plasmon Resonance Biosensor for Rapid Diagnosis of Severe Acute Respiratory Syndrome. Talanta.

[198] Zhang H., Harpster M.H., Wilson W.C., Johnson P.A. (2012). Surface-Enhanced Raman Scattering Detection of DNAs Derived from Virus Genomes Using Au-Coated Paramagnetic Nanoparticles. Langmuir.

[199] Singh R., Thakur P., Thakur A., Kumar H., Chawla P., Rohit J.V., Kaushik R., Kumar N. (2021). Colorimetric Sensing Approaches of Surface-Modified Gold and Silver Nanoparticles for Detection of Residual Pesticides: A Review. Int. J. Environ. Anal. Chem..

[200] Cao J., Galbraith E.K., Sun T., Grattan K.T.V. (2011). Comparison of Surface Plasmon Resonance and Localized Surface Plasmon Resonance-Based Optical Fibre Sensors. J. Phys. Conf. Ser..

[201] Takemura K., Adegoke O., Takahashi N., Kato T., Li T.-C., Kitamoto N., Tanaka T., Suzuki T., Park E.Y. (2017). Versatility of a Localized Surface Plasmon Resonance-Based Gold Nanoparticle-Alloyed Quantum Dot Nanobiosensor for Immunofluorescence Detection of Viruses. Biosens. Bioelectron..

[202] Inci F., Tokel O., Wang S., Gurkan U.A., Tasoglu S., Kuritzkes D.R., Demirci U. (2013). Nanoplasmonic Quantitative Detection of Intact Viruses from Unprocessed Whole Blood. ACS Nano.

[203] Pang Y., Rong Z., Wang J., Xiao R., Wang S. (2015). A Fluorescent Aptasensor for H5N1 Influenza Virus Detection Based-on the Core–Shell Nanoparticles Metal-Enhanced Fluorescence (MEF). Biosens. Bioelectron..

[204] Hu J., Jiang Y.-Z., Wu L.-L., Wu Z., Bi Y., Wong G., Qiu X., Chen J., Pang D.-W., Zhang Z.-L. (2017). Dual-Signal Readout Nanospheres for Rapid Point-of-Care Detection of Ebola Virus Glycoprotein. Anal. Chem..

[205] Driskell J.D., Shanmukh S., Liu Y.-J., Hennigan S., Jones L., Zhao Y.-P., Dluhy R.A., Krause D.C., Tripp R.A. (2008). Infectious Agent Detection With SERS-Active Silver Nanorod Arrays Prepared by Oblique Angle Deposition. IEEE Sens. J..

[206] Shanmukh S., Jones L., Driskell J., Zhao Y., Dluhy R., Tripp R.A. (2006). Rapid and Sensitive Detection of Respiratory Virus Molecular Signatures Using a Silver Nanorod Array SERS Substrate. Nano Lett..

[207] Driskell J.D., Zhu Y., Kirkwood C.D., Zhao Y., Dluhy R.A., Tripp R.A. (2010). Rapid and Sensitive Detection of Rotavirus Molecular Signatures Using Surface Enhanced Raman Spectroscopy. PLoS ONE.

[208] Sivashanmugan K., Liao J.-D., You J.-W., Wu C.-L. (2013). Focused-Ion-Beam-Fabricated Au/Ag Multilayered Nanorod Array as SERS-Active Substrate for Virus Strain Detection. Sens. Actuators B Chem..

[209] Sanjuán R. (2017). Collective Infectious Units in Viruses. Trends Microbiol..

[210] Saviñon-Flores F., Méndez E., López-Castaños M., Carabarin-Lima A., López-Castaños K.A., González-Fuentes M.A., Méndez-Albores A. (2021). A Review on SERS-Based Detection of Human Virus Infections: Influenza and Coronavirus. Biosensors.

[211] Lukose J., Chidangil S., George S.D. (2021). Optical Technologies for the Detection of Viruses like COVID-19: Progress and Prospects. Biosens. Bioelectron..

[212] Xu S., Ji X., Xu W., Li X., Wang L., Bai Y., Zhao B., Ozaki Y. (2004). Immunoassay Using Probe-Labelling Immunogold Nanoparticles with Silver Staining Enhancement via Surface-Enhanced Raman Scattering. Analyst.

[213] Jin Z., Du X., Xu Y., Deng Y., Liu M., Zhao Y., Zhang B., Li X., Zhang L., Peng C. (2020). Structure of Mpro from SARS-CoV-2 and Discovery of Its Inhibitors. Nature.

[214] Tai W., He L., Zhang X., Pu J., Voronin D., Jiang S., Zhou Y., Du L. (2020). Characterization of the Receptor-Binding Domain (RBD) of 2019 Novel Coronavirus: Implication for Development of RBD Protein as a Viral Attachment Inhibitor and Vaccine. Cell. Mol. Immunol..

[215] Ñique A.M., Coronado-Marquina F., Mendez Rico J.A., García Mendoza M.P., Rojas-Serrano N., Simas P.V.M., Cabezas Sanchez C., Drexler J.F. (2021). A Faster and Less Costly Alternative for RNA Extraction of SARS-CoV-2 Using Proteinase k Treatment Followed by Thermal Shock. PLoS ONE.

[216] Smyrlaki I., Ekman M., Lentini A., Rufino de Sousa N., Papanicolaou N., Vondracek M., Aarum J., Safari H., Muradrasoli S., Rothfuchs A.G. (2020). Massive and Rapid COVID-19 Testing Is Feasible by Extraction-Free SARS-CoV-2 RT-PCR. Nat. Commun..

[217] Gerber P.P., Duncan L.M., Greenwood E.J.D., Marelli S., Naamati A., Teixeira-Silva A., Crozier T.W.M., Gabaev I., Zhan J.R., Mulroney T.E. (2022). A Protease-Activatable Luminescent Biosensor and Reporter Cell Line for Authentic SARS-CoV-2 Infection. PLoS Pathog..

[218] COVID-19 Tests and Collection Kits Authorized by the FDA: Infographic | FDA. https://www.fda.gov/medical-devices/coronavirus-covid-19-and-medical-devices/covid-19-tests-and-collection-kits-authorized-fda-infographic.

[219] Afzal A. (2020). Molecular Diagnostic Technologies for COVID-19: Limitations and Challenges. J. Adv. Res..

[220] Antigen-Detection in the Diagnosis of SARS-CoV-2 Infection. https://www.who.int/publications/i/item/antigen-detection-in-the-diagnosis-of-sars-cov-2infection-using-rapid-immunoassays.

[221] Shen L., Zhang Q., Luo X., Xiao H., Gu M., Cao L., Zhao F., Chen Z. (2021). A Rapid Lateral Flow Immunoassay Strip for Detection of SARS-CoV-2 Antigen Using Latex Microspheres. J. Clin. Lab. Anal..

[222] Han W., Shin J.H. (2021). Low-Cost, Open-Source 3D Printed Antibody Dispenser for Development and Small-Scale Production of Lateral Flow Assay Strips. HardwareX.

[223] Pretti M.A.M., Galvani R.G., Scherer N.M., Farias A.S., Boroni M. (2022). In Silico Analysis of Mutant Epitopes in New SARS-CoV-2 Lineages Suggest Global Enhanced CD8+ T Cell Reactivity and Also Signs of Immune Response Escape. Infect. Genet. Evol..

[224] Zhang L., Jackson C.B., Mou H., Ojha A., Rangarajan E.S., Izard T., Farzan M., Choe H. (2020). The D614G Mutation in the SARS-CoV-2 Spike Protein Reduces S1 Shedding and Increases Infectivity. bioRxiv.

[225] Lubinski B., Fernandes M.H.V., Frazier L., Tang T., Daniel S., Diel D.G., Jaimes J.A., Whittaker G.R. (2021). Functional Evaluation of the P681H Mutation on the Proteolytic Activation the SARS-CoV-2 Variant B.1.1.7 (Alpha) Spike. bioRxiv.

[226] Starr T.N., Greaney A.J., Hilton S.K., Ellis D., Crawford K.H.D., Dingens A.S., Navarro M.J., Bowen J.E., Tortorici M.A., Walls A.C. (2020). Deep Mutational Scanning of SARS-CoV-2 Receptor Binding Domain Reveals Constraints on Folding and ACE2 Binding. Cell.

[227] Gobeil S.M.-C., Janowska K., McDowell S., Mansouri K., Parks R., Stalls V., Kopp M.F., Manne K., Li D., Wiehe K. (2021). Effect of Natural Mutations of SARS-CoV-2 on Spike Structure, Conformation, and Antigenicity. Science.

[228] Madhi S.A., Baillie V., Cutland C.L., Voysey M., Koen A.L., Fairlie L., Padayachee S.D., Dheda K., Barnabas S.L., Bhorat Q.E. (2021). Efficacy of the ChAdOx1 NCoV-19 Covid-19 Vaccine against the B.1.351 Variant. N. Engl. J. Med..

[229] Cele S., Gazy I., Jackson L., Hwa S.-H., Tegally H., Lustig G., Giandhari J., Pillay S., Wilkinson E., Naidoo Y. (2021). Escape of SARS-CoV-2 501Y.V2 from Neutralization by Convalescent Plasma. Nature.

[230] Lopez Bernal J., Andrews N., Gower C., Gallagher E., Simmons R., Thelwall S., Stowe J., Tessier E., Groves N., Dabrera G. (2021). Effectiveness of Covid-19 Vaccines against the B.1.617.2 (Delta) Variant. N. Engl. J. Med..

[231] Sheikh A., McMenamin J., Taylor B., Robertson C. (2021). SARS-CoV-2 Delta VOC in Scotland: Demographics, Risk of Hospital Admission, and Vaccine Effectiveness. Lancet.

[232] Jangra S., Ye C., Rathnasinghe R., Stadlbauer D., Krammer F., Simon V., Martinez-Sobrido L., Garćia-Sastre A., Schotsaert M., PVI Study Group (2021). The E484K Mutation in the SARS-CoV-2 Spike Protein Reduces but Does Not Abolish Neutralizing Activity of Human Convalescent and Post-Vaccination Sera. medRxiv.

[233] Baum A., Fulton B.O., Wloga E., Copin R., Pascal K.E., Russo V., Giordano S., Lanza K., Negron N., Ni M. (2020). Antibody Cocktail to SARS-CoV-2 Spike Protein Prevents Rapid Mutational Escape Seen with Individual Antibodies. Science.

[234] Jangra S., Ye C., Rathnasinghe R., Stadlbauer D., Alshammary H., Amoako A.A., Awawda M.H., Beach K.F., Bermúdez-González M.C., Chernet R.L. (2021). SARS-CoV-2 Spike E484K Mutation Reduces Antibody Neutralisation. Lancet Microbe.

[235] Cov-Lineages. https://cov-lineages.org/.

[236] O’Toole Á., Scher E., Underwood A., Jackson B., Hill V., McCrone J.T., Colquhoun R., Ruis C., Abu-Dahab K., Taylor B. (2021). Assignment of Epidemiological Lineages in an Emerging Pandemic Using the Pangolin Tool. Virus Evol..

[237] O’Toole Á., Kraemer M.U.G., Hill V., Pybus O.G., Watts A., Bogoch I.I., Khan K., Messina J.P., Tegally H., Lessells R.R. (2021). Tracking the International Spread of SARS-CoV-2 Lineages B.1.1.7 and B.1.351/501Y-V2. Wellcome Open Res..

[238] Rambaut A., Holmes E.C., O’Toole Á., Hill V., McCrone J.T., Ruis C., du Plessis L., Pybus O.G. (2020). A Dynamic Nomenclature Proposal for SARS-CoV-2 Lineages to Assist Genomic Epidemiology. Nat. Microbiol..

[239] Davies N.G., Jarvis C.I., van Zandvoort K., Clifford S., Sun F.Y., Funk S., Medley G., Jafari Y., Meakin S.R., Lowe R. (2021). Increased Mortality in Community-Tested Cases of SARS-CoV-2 Lineage B.1.1.7. Nature.

[240] Davies N.G., Abbott S., Barnard R.C., Jarvis C.I., Kucharski A.J., Munday J.D., Pearson C.A.B., Russell T.W., Tully D.C., Washburne A.D. (2021). Estimated Transmissibility and Impact of SARS-CoV-2 Lineage B.1.1.7 in England. Science.

[241] Funk T., Pharris A., Spiteri G., Bundle N., Melidou A., Carr M., Gonzalez G., Garcia-Leon A., Crispie F., O’Connor L. (2021). Characteristics of SARS-CoV-2 Variants of Concern B.1.1.7, B.1.351 or P.1: Data from Seven EU/EEA Countries, Weeks 38/2020 to 10/2021. Eurosurveillance.

[242] Pereira F., Tosta S., Lima M.M., de Oliveira da Silva L., Nardy V.B., Gómez M.K.A., Lima J.G., Fonseca V., de Oliveira T., Lourenço J. (2021). Genomic Surveillance Activities Unveil the Introduction of the SARS-CoV-2 B.1.525 Variant of Interest in Brazil: Case Report. J. Med. Virol..

[243] Ozer E.A., Simons L.M., Adewumi O.M., Fowotade A.A., Omoruyi E.C., Adeniji J.A., Dean T.J., Zayas J., Bhimalli P.P., Ash M.K. (2021). Coincident Rapid Expansion of Two SARS-CoV-2 Lineages with Enhanced Infectivity in Nigeria. medRxiv.

[244] Nonaka C.K.V., Gräf T., de Lorenzo Barcia C.A., Costa V.F., de Oliveira J.L., da Hora Passos R., Bastos I.N., de Santana M.C.B., Santos I.M., de Sousa K.A.F. (2021). SARS-CoV-2 Variant of Concern P.1 (Gamma) Infection in Young and Middle-Aged Patients Admitted to the Intensive Care Units of a Single Hospital in Salvador, Northeast Brazil, February 2021. Int. J. Infect. Dis..

[245] Bugembe D.L., Phan M.V.T., Ssewanyana I., Semanda P., Nansumba H., Dhaala B., Nabadda S., O’Toole Á.N., Rambaut A., Kaleebu P. (2021). A SARS-CoV-2 Lineage A Variant (A.23.1) with Altered Spike Has Emerged and Is Dominating the Current Uganda Epidemic. medRxiv.

[246] Elaissari A., Holt L., Meunier F., Voisset C., Pichot C., Mandrand B., Mabilat C. (1999). Hydrophilic and Cationic Latex Particles for the Specific Extraction of Nucleic Acids. J. Biomater. Sci. Polym. Ed..

[247] Sun N., Deng C., Liu Y., Zhao X., Tang Y., Liu R., Xia Q., Yan W., Ge G. (2014). Optimization of Influencing Factors of Nucleic Acid Adsorption onto Silica-Coated Magnetic Particles: Application to Viral Nucleic Acid Extraction from Serum. J. Chromatogr. A.

[248] Dighe K., Moitra P., Alafeef M., Gunaseelan N., Pan D. (2022). A Rapid RNA Extraction-Free Lateral Flow Assay for Molecular Point-of-Care Detection of SARS-CoV-2 Augmented by Chemical Probes. Biosens. Bioelectron..

[249] Bruno A., de Mora D., Freire-Paspuel B., Rodriguez A.S., Paredes-Espinosa M.B., Olmedo M., Sanchez M., Romero J., Paez M., Gonzalez M. (2021). Analytical and Clinical Evaluation of a Heat Shock SARS-CoV-2 Detection Method without RNA Extraction for N and E Genes RT-QPCR. Int. J. Infect. Dis..

[250] Chomczynski P., Sacchi N. (2006). The Single-Step Method of RNA Isolation by Acid Guanidinium Thiocyanate–Phenol–Chloroform Extraction: Twenty-Something Years On. Nat. Protoc..

[251] Chomczynski P., Sacchi N. (1987). Single-Step Method of RNA Isolation by Acid Guanidinium Thiocyanate-Phenol-Chloroform Extraction. Anal. Biochem..

[252] He H., Li R., Chen Y., Pan P., Tong W., Dong X., Chen Y., Yu D. (2017). Integrated DNA and RNA Extraction Using Magnetic Beads from Viral Pathogens Causing Acute Respiratory Infections. Sci. Rep..

[253] Pan Y., Long L., Zhang D., Yuan T., Cui S., Yang P., Wang Q., Ren S. (2020). Potential False-Negative Nucleic Acid Testing Results for Severe Acute Respiratory Syndrome Coronavirus 2 from Thermal Inactivation of Samples with Low Viral Loads. Clin. Chem..

[254] Nyan D.-C.C., Ulitzky L.E., Cehan N., Williamson P., Winkelman V., Rios M., Taylor D.R. (2014). Rapid Detection of Hepatitis B Virus in Blood Plasma by a Specific and Sensitive Loop-Mediated Isothermal Amplification Assay. Clin. Infect. Dis..

[255] Pan L., Mu M., Yang P., Sun Y., Wang R., Yan J., Li P., Hu B., Wang J., Hu C. (2020). Clinical Characteristics of COVID-19 Patients With Digestive Symptoms in Hubei, China: A Descriptive, Cross-Sectional, Multicenter Study. Off. J. Am. Coll. Gastroenterol. | ACG.

[256] Perumal N., Jain R., Shrivastava R., Lalwani J., Chaurasia D. (2020). Stability of SARS-CoV-2 RNA in Viral Lysis Buffer Stored at Different Temperatures. J. Lab. Physicians.

[257] Wozniak A., Cerda A., Ibarra-Henríquez C., Sebastian V., Armijo G., Lamig L., Miranda C., Lagos M., Solari S., Guzmán A.M. (2020). A Simple RNA Preparation Method for SARS-CoV-2 Detection by RT-QPCR. Sci. Rep..

[258] Barza R., Patel P., Sabatini L., Singh K. (2020). Use of a Simplified Sample Processing Step without RNA Extraction for Direct SARS-CoV-2 RT-PCR Detection. J. Clin. Virol..

[259] Ranoa D.R.E., Holland R.L., Alnaji F.G., Green K.J., Wang L., Brooke C.B., Burke M.D., Fan T.M., Hergenrother P.J. (2020). Saliva-Based Molecular Testing for SARS-CoV-2 That Bypasses RNA Extraction. bioRxiv.

[260] Mauriz E. (2020). Clinical Applications of Visual Plasmonic Colorimetric Sensing. Sensors.

[261] Nelson M. (2021). Biosphere 2’s Lessons about Living on Earth and in Space. Sp. Sci. Technol..

[262] Cinelli I., Russomano T. (2021). Advances in Space Medicine Applied to Pandemics on Earth. Sp. Sci. Technol..

